# Gene expression and evolution of Bowman-Birk protease inhibitors in wild and domesticated *Vigna* (Fabaceae) species

**DOI:** 10.3389/fpls.2025.1657741

**Published:** 2026-01-28

**Authors:** Elisa Toini, Maria Totaro, Giuseppe Silvestri, Jacopo Vertemara, Giovanni Zecca, Davide Panzeri, Emily Rose Palm, Robert Philipp Wagensommer, Giuseppe Zampella, Massimo Labra, Fabrizio Grassi

**Affiliations:** 1 Department of Biotechnology and Bioscience, University of Milan-Bicocca, Milano, Italy; 2 National Biodiversity Future Center (NBFC), Palermo, Italy; 3 Faculty of Education, Free University of Bozen-Bolzano, Bolzano, Italy

**Keywords:** binding energy, bioprospecting, Bowman-Birk protease inhibitors, Fabaceae, gene diversity, Vigna

## Abstract

Bowman-Birk protease inhibitors (BBIs) are multifunctional proteins with a double-headed structure, featuring two distinct inhibitory loops that target trypsin and chymotrypsin proteases. BBI regulates protease activity in plants and provides defense against pests and pathogens, but little is still known about their expression levels and their ability to interact with natural targets. Our results showed that *BBI1* and *BBI2* genes are the most highly expressed in *Vigna* seeds. Consequently, we produced two multiple sequence alignments including homologs from 42 *Vigna* taxa to explore variability and functionality. Phylogenetic relationships, signals of positive selection, and interaction energy levels with their natural targets were inferred. Overall, BBI2 exhibited the highest affinity for the assessed targets compared to BBI1. Amino acid substitutions have led to distinct protein variants across species, each displaying different interaction capacities with their respective targets. Additionally, the residue conferring inhibitory specificity for trypsin, located in the first domain, was found to be under positive selection in both genes. This suggests an ongoing evolutionary process aimed at optimizing affinity with proteases through continuous adaptation. Finally we emphasize that findings obtained can be used to drive the activity of plant breeders and more efficient cultivars can be selected. Given the growing availability of genomic information of wild and domesticated accessions, docking simulations offer a convenient and effective method to preliminarily assess new protein variants.

## 1 Introduction

Bowman-Birk protease inhibitors (BBIs) are a family of serine-type protease inhibitors that play a pivotal role in plant development and defenses (Bowman, 1946; Shan et al., 2008; Dramé et al., 2013; Malefo et al., 2020; Xie et al., 2021). Recent phylogenetic analyses suggest that angiosperms share a common ancestral BBI inhibitory loop with early vascular plants that appeared in the lycopod plant lineage 370 million years ago (James et al., 2017). In modern plants, BBI-type inhibitors are mainly distributed in the Poaceae and Leguminosae families. In legumes, they possess a double inhibitory-loop structure, which was lost in the monocot lineage due to the loss of two cysteine residues, resulting in a single-headed structure (James et al., 2017). In Fabaceae, BBIs are composed of about 70 amino acids that fold to expose hydrophobic patches but lack a hydrophobic core. This structure is highly stable under different conditions, such as high temperature and a wide pH range, due to seven disulfide bridges, and forms a double-headed configuration with two independent inhibitory domains (Odani and Ikenaka, 1973; Domoney, 1999; Mello et al., 2003; Barbosa et al., 2007; Losso, 2008). Classified as I12 family in the MEROPS database (Rawlings et al., 2014)^1^, these inhibitors share a binding loop motif where P1 residues serve as primary contact residues defining the interaction with the target enzyme (**Supplementary Figure S1**) (Schechter and Berger, 1967; Grosse-Holz and van der Hoorn, 2016). Different classes of BBI proteins, which arose from an ancestral duplication of a single-headed inhibitor gene, have demonstrated the ability to inhibit trypsin (where both reactive sites inhibit trypsin) and chymotrypsin (where the first reactive site inhibits trypsin and the second inhibits chymotrypsin). Furthermore, some studies suggest that BBI also reduces the proteolytic activities of other proteases such as elastase, cathepsin G, and chymase (Clemente and Domoney, 2006; Losso, 2008; De Paola et al., 2012; Ferreira et al., 2019).

Legume seeds are a rich source of BBIs and their activity suppresses pest and pathogen digestion, playing a defensive role conferring resistance in response to wounding by insects (e.g. Coleoptera and Lepidoptera) in different plant species (Hilder et al., 1987; Rakwal et al., 2001; Qu et al., 2003) Moreover, BBIs exhibit *in vitro* antifungal activity against different pathogens such as *Botrytis cinerea* and *Fusarium culmorum* (Ye et al., 2001; Pekkarinen et al., 2007) and appear to be involved in diverse biological processes including drought stress (Yan et al., 2009; Malefo et al., 2020) and tolerance to salinity (Shan et al., 2008).

In the last decade, BBIs have received a growing interest, and epidemiological studies have shown that diets based on legumes have a positive impact on human health (Sánchez-Chino et al., 2015; de Freitas et al., 2020; Carbonaro and Nucara, 2022). Although the underlying mechanism is not yet fully understood, the capacity to control the cellular growth and proliferation of some cancers (e.g. colorectal, prostate, ovarian, oral cavity) as well as the ability to suppress chronic inflammations of different origin have been widely reported (Clemente et al., 2005; Chen et al., 2005; Clemente and del Carmen Arques, 2014; Juritsch and Moreau, 2018; Gitlin-Domagalska et al., 2020). Some studies suggest that their beneficial effects are due to the inhibition of the proteasome, related to anticancer activity, and to the inhibition of proteases produced by macrophages and mast cells during inflammation, such as chymase, neutrophil elastase, and cathepsin G, directly supporting their anti-inflammatory properties (Gitlin-Domagalska et al., 2020). BBI targets are primarily members of the S1 and S3 peptidase families, and interactions with these enzymes are likely responsible for many of their biological properties (Rawlings et al., 2014)^2^.

In recent years, a growing number of legumes have been analyzed with encouraging results showing that BBIs extracted from cowpea (*Vigna unguiculata* L.) seeds are good candidates for the treatment of various pathological states such as neurodegeneration, hypertension and different types of cancers such as breast, colorectal, and prostate (Joanitti et al., 2010; Souza et al., 2014; Panzeri et al., 2020; Gitlin-Domagalska et al., 2020). However, analyses of BBI expression levels as well as a general characterization of BBI genes in the genus *Vigna* have not yet been performed. This lack of information is surprising, given both the extent to which many communities around the world are reliant on both domesticated and wild lines of *Vigna* (Maxted et al., 2004; Nair et al., 2023), and the potential health benefits already observed from BBIs isolated from cowpea (Joanitti et al., 2010; Mehdad et al., 2016; Panzeri et al., 2020).

The genus *Vigna* (Fabaceae) includes over 100 species (Plants of the World Online, 2025), many of which are cultivated in tropical and subtropical regions (Tomooka et al., 2002; Maxted et al., 2004), with cowpea (*V. unguiculata*) having the largest cultivation area (Kebede and Bekeko, 2020). These species are highly adaptable to extreme environments, such as drought, poor soils, and high temperatures, making them valuable for subsistence agriculture (Nair et al., 2023). They provide food, forage, and other benefits, while wild relatives serve as genetic resources for improving stress tolerance, disease resistance, and agronomic traits, supporting the development of resilient varieties under climate change (Sharma et al., 2013; Dwivedi et al., 2016; Kumari et al., 2021). Furthermore, beans are widely consumed by African and Asian communities, who integrate these legumes into an expanding variety of traditional and modern dishes known for their affordability and sustainability (Wang et al., 2022; Singh et al., 2022; Kanishka et al., 2023; Harouna et al., 2024).

To further our understanding of the distribution of BBI genes within the genus *Vigna*, we used a multidisciplinary genetic, phylogenetic and computational approach and investigated their expression levels in a number of carefully selected domesticated and wild lines that are representative of their geographic and environmental distribution. Given the high adaptability of this genus to a wide variety of edaphic conditions (Nair et al., 2023) and the role of BBI proteins in plant defenses to both biotic and abiotic stresses (Clemente and Domoney, 2006), we hypothesized that there would also be a high degree of genetic variability in terms of BBI gene expression across the genus.

BBIs have been isolated and described in various legumes, however, the expression levels as well as the characterization of BBI genes in *Vigna* genus is lacking. In this study, we retrieve all the BBIs genes present in 12 *Vigna* species genomes. Then we conducted a preliminary analysis of the expression of BBI genes in five *Vigna* species in both leaves and seeds, as these tissues are highly relevant to both plant fitness and human nutrition. The two BBI genes mainly expressed in seeds were successively sequenced in 42 *Vigna* taxa, including both crops and wild relatives. The levels of interaction energy with the main human targets (trypsin and chymotrypsin) are calculated for each mature protein and the results are discussed in an evolutionary context. This study allowed us to gain deeper insight into the expression, natural variability, evolution and property of BBI genes in the genus *Vigna*, particularly in seeds, providing valuable information for the development of future crops.

## 2 Materials and methods

### 2.1 BBI genes identification

BBI genes analysed in this study were identified from a comparison of 12 different *Vigna* genomes, including both domesticated and wild species originating from Africa and Asia, by exploring three different databases (**Supplementary Table S1**). To achieve this, the sequence of the first inhibitory domain of BBI known to inhibit trypsin obtained by *Vigna unguiculata* was blasted using the tblastx algorithm to find the position of the BBIs genes in the genome. The entire gene was then retrieved from the surrounding sequence. The BBI genes were analyzed using OrthoFinder 2.5.5 with default parameters (Emms and Kelly, 2019) to identify orthologous and paralogous genes. A multi alignment was generated using MAFFT v. 7 (Katoh et al., 2019), then ModelFinder and IQ-TREE were used respectively to select the best-fitting substitution models and to produce the Maximum Likelihood tree (ML-tree) (Kalyaanamoorthy et al., 2017; Minh et al., 2020). The analysis was performed using the TIM3+F+I+G4 evolutionary model and including 1000 ultrafast bootstrap replicates.

### 2.2 Selection of samples

For RNA expression analysis, five species were selected from 12 *Vigna* genomes previously analyzed for BBI gene identification: five wild accessions (*V. unguiculata ssp. dekindtiana* (L.) Walp., *V. marina* (Burm.f.) Merr., *V. mungo* (L.) Hepper, *V. radiata* (L.) R.Wilczek and *V. vexillata* (L.) A. Rich) and 2 domesticated accessions (*V. unguiculata ssp. unguiculata* (L.) Walp. and *V. radiata* (L.) R. Wilczek). These species were chosen based on their relevance to human nutrition and their geographical distribution across the two main continents where the genus occurs. The Asian representatives were *V. radiata* and *V. mungo*, while the African representatives were *V. unguiculata*, *V. marina*, and *V. vexillata*. The origins of individual samples are given in **Supplementary Figure S2** and **Supplementary Table S2**. Successively, a total of 95 individuals belonging to 42 species of *Vigna* genus, covering both African and Asian taxa, were selected (origin of samples are given in **Supplementary Table S3**) to explore the variability of *BBI1* and *BBI2* genes.

### 2.3 RNA extraction, cDNA synthesis, RT-qPCR amplification

We based our protocol on the gene expression methodologies reported in other studies (Vu et al., 2021; Al-Dulaimi et al., 2022; Gong et al., 2022). Seeds and leaves were chosen as target organs to assess RNA expression. Leaf samples were obtained from fully developed first unifoliate leaves of plants grown as follows. First the seeds were washed with 10% bleach for about one minute and then thoroughly rinsed with distilled water. Imbibition was stimulated by gentle abrasion of the seed coat, and the seeds were placed onto wet paper to promote germination. The sprouts were grown for about 15 days until the first two unifoliate leaves were completely developed (Hu et al., 2009; Paul et al., 2014). At harvest the seedlings were no taller than 20 cm. RNA was extracted from six samples for each accession, comprised of three seed samples and leaf samples from three individual seedlings. For all samples, RNA extraction was performed using the RNeasy Plant Mini Kit (Qiagen) following the manufacturer’s protocol. Proprietary RLC buffer was chosen over RLT because of the presence of secondary metabolites, confirmed during the set-up phase with test samples. All the procedure was performed keeping the temperature below 15 °C using ice and cooled tubes. Samples were quantified by NanoDrop One (ThermoFisher) and subsequently stored at -80 °C (main details of RNA extraction are reported in appendix). For cDNA synthesis, 10 μl of RNA (about 2-3 μg) was mixed with 10 μl of ZymoScript RT PreMix Kit (ZymoResarch). The reaction mixture was then put in a Swift-MaxPro (Esco Healthcare) thermocycler with the following program: 2 minutes of incubation at 25 °C, 30 minutes of extension at 42 °C to extend even long RNAs, 1 minute of enzyme inactivation at 95 °C. cDNA was then stored at -20 °C. To evaluate gene expression, a Real Time PCR approach was used (StepOnePlus, ThermoFisher). cDNA solutions were prepared by diluting the cDNA with sterile MilliQ water to reach a fixed concentration of 10 ng/μl for each sample. Then the reaction mixture was set up as follows: 1 μl (10 ng/μl) of cDNA, 10 μl of Luna qPCR Universal Master Mix (New England Biolabs), 0.5 μl of each specific primer, 8 μl of Sterile MilliQ Water (Real Time PCR protocol and list of primers are described in appendix and **Supplementary Table S4**). Run data was then processed with StepOne Software (ThermoFisher, v2.3). Ct threshold was fixed at Ct=0.4 and actin was chosen as the reference gene (Costa et al., 2010). Actin normalized data, expressed as -ΔCt (Ctactin - Cttarget), were used to perform analyses. To detect expression differences of the same BBI gene in different organs (seeds vs. leaves) a t-test was chosen. To determine the expression contribution of all BBI genes in the same organ (seeds or leaves) an ANOVA test coupled with Tukey-HSD *post-hoc* analysis was performed. The t-test was performed in Excel exploiting the Analysis Tool-Pak add-in (v. 2402 Build 16.0.17328.20124), while ANOVA analysis was performed in R Studio IDE (ver. 2023.09.1 Build 494). Graphic outputs were generated using the ggplot2 R package (Hadley, 2016).

### 2.4 Genomic DNA extraction, PCR-amplification, sequencing and alignment

The genomic DNA of 95 samples was extracted from leaves using the E.Z.N.A.^®^ Plant DNA kit (Omega Bio-Tek). This involved first the mechanical crushing of samples, soaking for a few minutes in liquid nitrogen with zirconia/silica beads (2, 5 mm) in Mini Bead Beater (Biospec Products) and then chemical and thermal lysis. After precipitation of polysaccharides and elimination of RNA, DNA was eluted to obtain the final sample. *BBI1* and *BBI2* genes were amplified using Esco Healthcare Swift-MaxPro thermocycler. Lists of primers used in this study to amplify and to sequence both genes are reported in **Supplementary Table S5** and **Supplementary Table S6**. The total reaction volume of 25 μL included 1 µl of genomic DNA, 12.5 μLGoTaq^®^ G2 Green Master Mix (Promega), 0.5 µl of each primer and purified water until the final reaction volume. The PCR reaction required an initial temperature of 95 °C for 5 minutes for denaturation, then 35 amplification cycles each with 30 seconds of denaturation at 95 °C, 35 seconds of annealing using a range of temperature from 48 °C to 58 °C, 45 seconds of extension at 72 °C and finally 2 minutes to complete extension at 72 °C. Successively, products of PCR were visualized by 2% agarose gel electrophoresis stained with EuroSafe Nucleic Acid Stain (EuroClone) and purified using the QIAquick PCR Purification Kit (Qiagen). DNA sequencing was performed by Eurofins Genomics Srl (Vimodrone, Milano, Italy) and Macrogen Europe (Milano, Italy) and all DNA sequences were verified visually using BioEdit 7.2.5 (Hall, 1999) program. Positions in the electropherogram displaying overlapping peaks at the same nucleotide, caused either by heterozygosity or by recent gene copies, were coded using the IUPAC degenerate nucleotide codes. Finally, the BBI1 and BBI2 sequences previously identified from the *Vigna* genomes, along with 4 sequences from NCBI, were added, and the online version of MAFFT v. 7 (Katoh et al., 2019) was used to generate multiple sequence alignments for *BBI1* and *BBI2* genes.

### 2.5 Molecular dating

Divergence times were estimated using BEAST 2.6.7 (Bouckaert et al., 2014) and assumed the Yule speciation model and the molecular strict clock for both data sets. Moreover, the HKY+G substitution model for the *BBI1* gene and GTR+G substitution model for the *BBI2* gene were selected as estimated by the jModelTest 2.1.10 (Darriba et al., 2012). Due to the scarcity of fossil information for *Vigna*, we opted to use secondary calibration by integrating information from multiple sources. Li et al. (2013) estimate the divergence between *Vigna* and *Lablab* at 11.2 million years ago (Mya), and between *Phaseolus* and *Vigna* at 9.7 mya. However, Chang et al. (2019) report a divergence between *Vigna* and *Lablab* at 12.5 Mya and between *Phaseolus* and *Vigna* at 10.2 Mya. Given this uncertainty, we decided to set our calibrations using broad Highest Posterior Density (HPD) intervals. A normal distribution with the mean of 12 Mya and a standard deviation of 1.5 (95% HPD interval of 14.09–9.06 Mya) was assumed as prior on the root and a normal distribution with the mean of 10 Mya and a standard deviation of 1.0 (95% HPD interval of 12.00–8.04 Mya) was assumed as prior on the split between *Vigna* and *Phaseolus* (Li et al., 2013; Chang et al.). Two independent MCMC chains were run for 50 million generations, and the convergence of runs was checked using Tracer 1.7^3^ (http://beast.bio.ed.ac.uk/Tracer). All parameters have shown an Effective Sample Size (ESS) exceeding 200. Outputs were combined with LogCombiner and an annotated maximum clade credibility tree that shows median node ‘heights’ was generated with TreeAnnotator (both software are implemented in Beast). The final trees were visualized in FigTree 1.4.3^4^.

### 2.6 Positive selection

The Site Model test was conducted for the *BBI1* and *BBI2* genes using the CODEML algorithm (Yang, 1997) with the clean data option, and a likelihood ratio test was applied to detect positive selection (M8a vs. M8 models). To identify codons under positive selection we performed FUBAR and MEME analysis, implemented in DATAMONKEY web interface, and successively NEB and BEB implemented in EasyCodeML (Murrell et al., 2013; Weaver et al., 2018; Gao et al., 2019). FUBAR was run using the default option and the following advanced options: number of grid points = 40; concentration parameter of the Dirichlet prior = 0.5. Codons under positive selection were reported when FUBAR, NEB and BEB analyses showed P ≥ 0.95 and when MEME analysis showed p ≤ 0.05.

### 2.7 Inferring ancestral amino acid sequences reconstruction

IQ-TREE and ModelFinder were used respectively to produce the ML-trees and to select the best-fitting scheme of substitution models (Kalyaanamoorthy et al., 2017; Minh et al., 2020). In the tree search, we assumed 100 initial parsimony trees to optimize with the NNI (nearest neighbor interchange) search to initialize the candidate set. To infer the ML-trees we used the following setting: evolutionary model = JTT+G, perturbation strength = 0.2 and stopping rule = 500. To assess significance of nodes, we applied the Shimodara-Hasegawa-like approximate likelihood ratio test (SH-aLRT) with 5000 replicates, the Approximate Bayes Test and Ultrafast bootstrap with 5000 replicates. Ancestral sequence reconstruction (ASR) methods are used to identify the ancestral isoforms (AncI) and ancestral proteins (AncP) using GRASP (Foley et al., 2022). The software uses isoform alignments and ML-trees as input data and we have chosen to infer the most likely state by Marginal and Joint reconstruction methods, setting the JTT model for both alignments.

### 2.8 Binding energy calculations

For the docking simulations, human variants of trypsin and chymotrypsin were used, as BBIs are known for their ability to regulate cell growth and proliferation in certain types of cancer by inhibiting trypsin- and chymotrypsin-like proteases (Clemente and del Carmen Arques, 2014). The structures of human trypsin and human chymotrypsin were retrieved from the Protein Data Bank, 1TRN and 4CHA, respectively. The crystallographic structures were processed with Maestro using Protein Preparation Wizard (Sastry et al., 2013) to reconstruct any missing loops or unresolved residues, assign the correct bond order, create disulfide bridges and generate the correct protonation state of the residues at pH 7.0. Structures of the BBI mature proteins were predicted using AlphaFold v2.0 (Jumper et al., 2021), plDDT values are reported in the **Supplementary Materials** (**Supplementary Figure S4**). Docking simulations were performed using HADDOCK3^5^ targeting the binding regions of selected BBI inhibitors. For each BBI-trypsin and BBI-chymotrypsin complex, the HADDOCK protocol involves three sequential steps: i) full randomization of the orientations and docking by rigid-body energy minimization; ii) semi-flexible refinement by simulated annealing in torsion angle space during which the interfaces are considered flexible; iii) refinement by a short molecular dynamics simulation in explicit solvent and finally an energy minimization. All the generated structures were clustered according to the fraction of common contacts (FCC). Structures from the cluster with the best docking score were selected to predict ΔG using the PRODIGY predictor (Vangone and Bonvin, 2017).

## 3 Results

### 3.1 BBI genes identification

A total of 50 complete genes were identified in 12 *Vigna* genomes. The number of genes identified was different for each species, ranging from 2 in *V. radiata* to 7 in *V. unguiculata*. OrthoFinder analysis classified these 50 genes into four distinct orthogroups (*BBI1*, *BBI2*, *BBI3*, *BBI4*). A graphical representation of relationships between genes is shown in **Supplementary Figure S3**. Going forward, each gene from a different orthogroup is followed by a number, and multiple genes from the same species within an orthogroup are also followed by a letter. The identification of BBI genes within genomes enabled the design of primers used in successive analysis.

### 3.2 Gene expression

**Figure 1:**
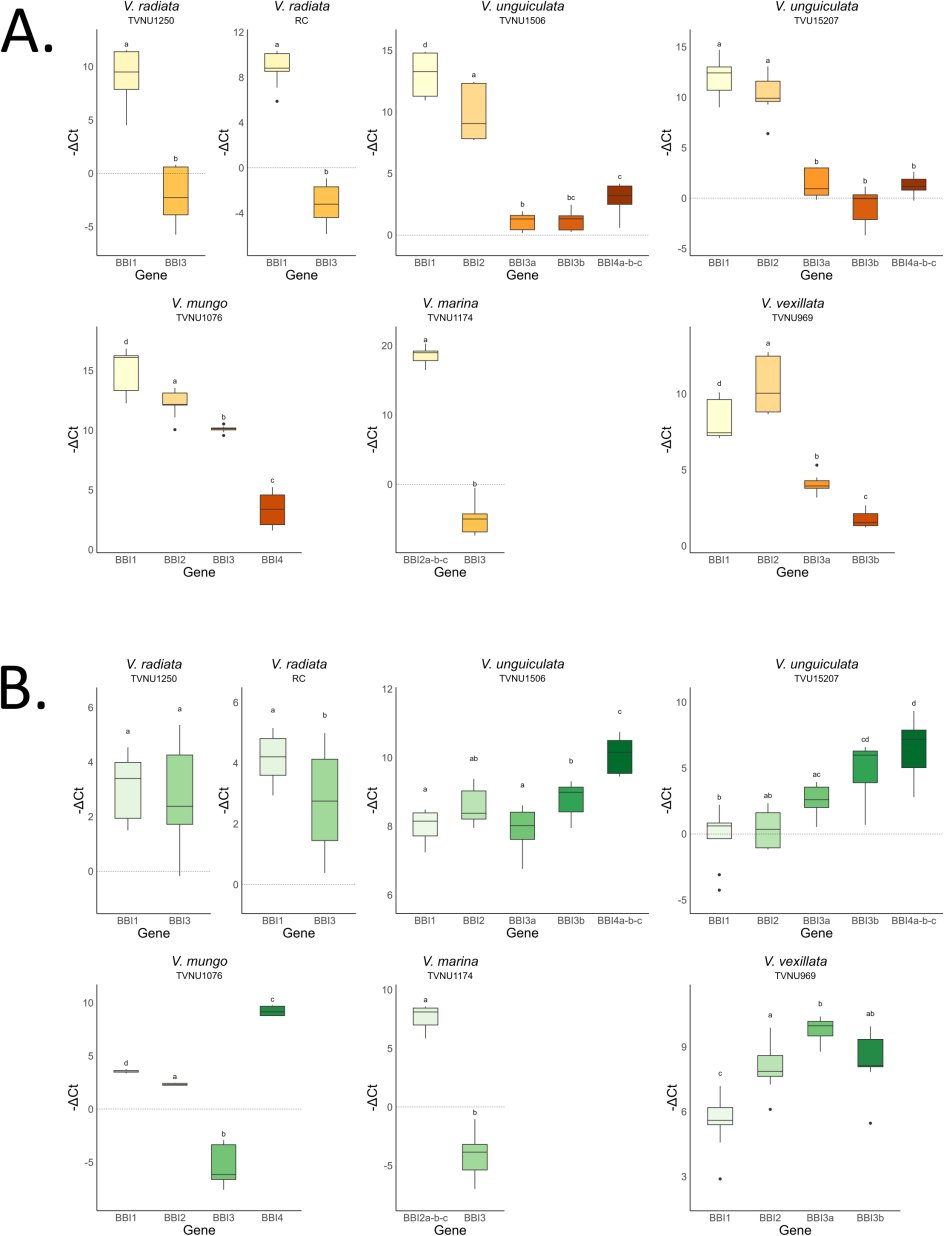
Comparison of expression level among BBI genes observed in seeds **(A)** and leaves **(B)** collected from 5 different *Vigna* species. On the on the x-axis are the different BBI genes, each gene is named based on the orthogroups (*BBI1*, *BBI2*, *BBI3*, *BBI4*) and multiple genes from the same species within an orthogroup are also followed by a letter. On the on the y-axis -ΔCt (Ctactin - Cttarget) represents the value of the expression levels normalized to the housekeeping gene actin. Different lowercase letters over the boxes indicate significant differences (p < 0.05) and points indicate the outlier values. In two cases (*V. unguiculata BBI4*a-b-c, *V. marina BBI2*a-b-c) primers have amplified more than one gene.

The expression levels of BBI genes analyzed across five species are shown in **Figure 1**. In seeds, the *BBI1* and *BBI2* genes consistently exhibit significantly higher expression levels compared to the other genes (**Figure 1**). Additionally, these two genes are expressed at higher levels in seeds than in leaves (**Supplementary Table S7**). However, it is important to note that, in some instances, the primers used in this study amplified multiple genes, as it was not feasible to target individual genes. As a result, this may have led to an overestimation of expression levels in those cases.

### 3.3 Phylogenetic analysis, divergence times and natural selection

**Figure 2:**
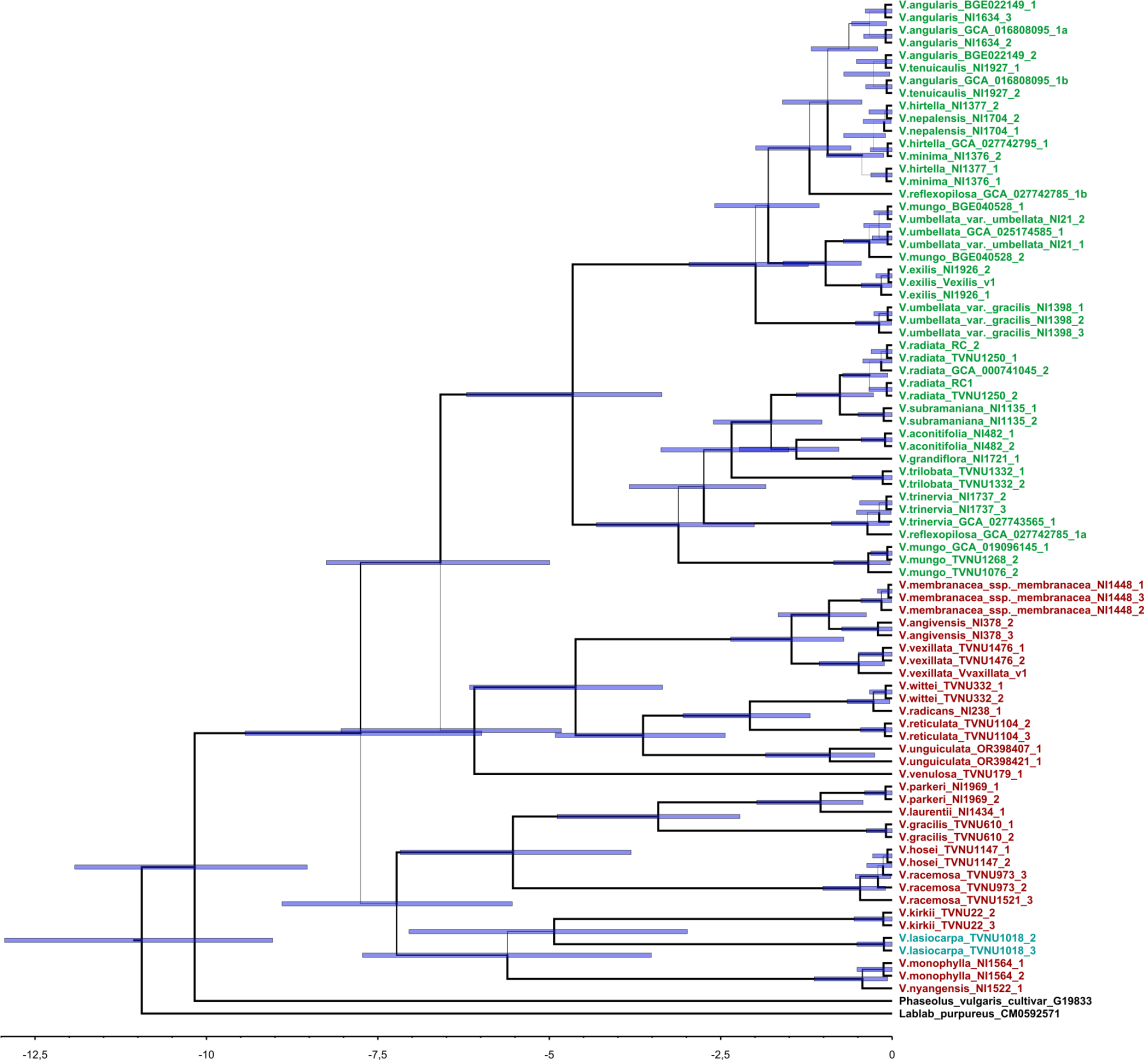
Maximum clade credibility tree computed by BEAST 2.6.7 based on *BBI1* (BBI genes part of the *BBI1* orthogroup) gene alignment. Colors of names represent the origin taxa, green = Asia, red = Africa, Blu = America. Line width is proportional to Bayesian posterior probabilities whereas blue bars represent the associated credibility interval (95% HPD). The scale at the bottom of the figure is reported in millions of years before the present.

**Figure 3:**
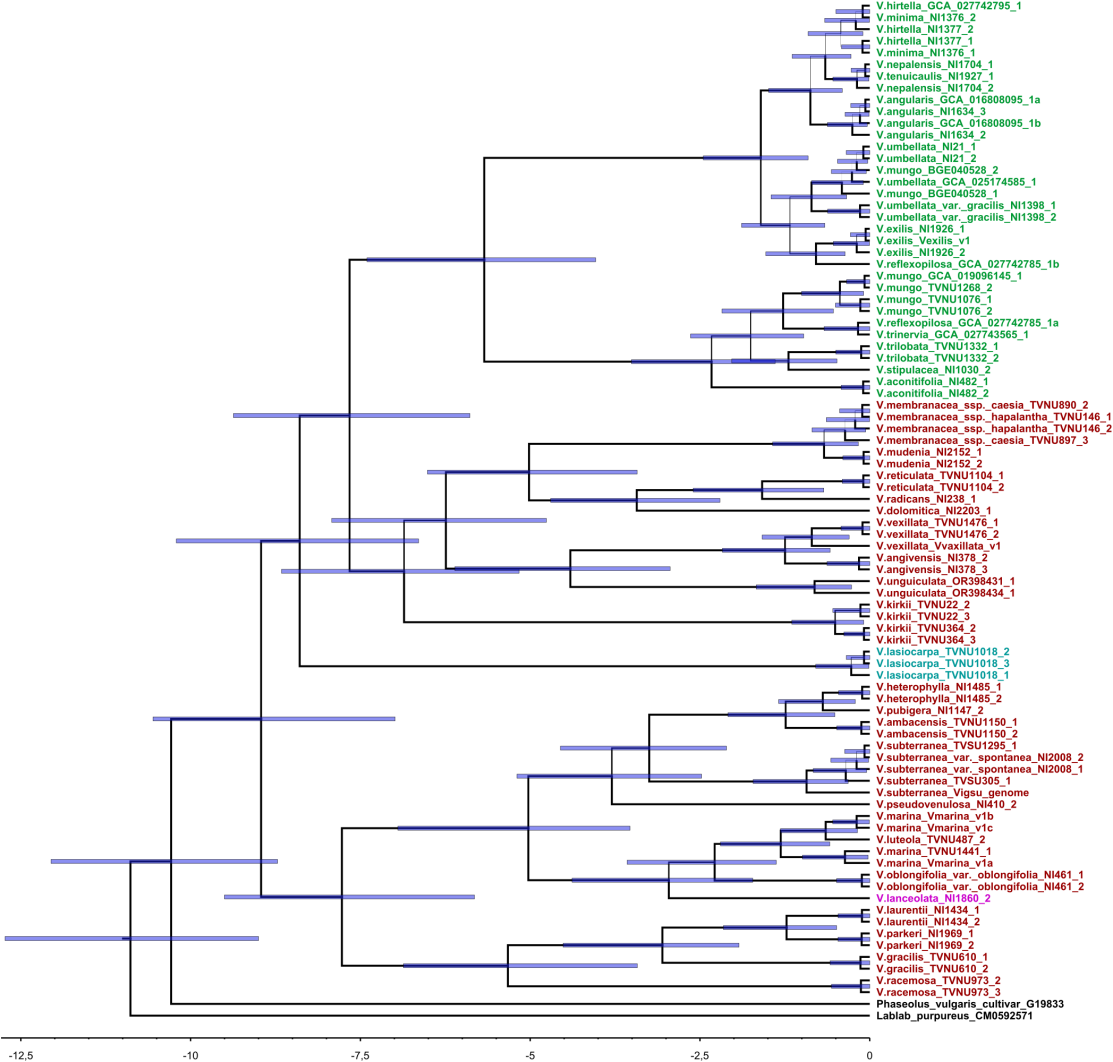
Maximum clade credibility tree computed by BEAST 2.6.7 based on *BBI2* (BBI genes part of the *BBI2* orthogroup) gene alignment. Colors of names represent the origin of taxa, green = Asia, red = Africa, Blu = America, Purple = Oceania. Line width is proportional to Bayesian posterior probabilities whereas blue bars represent the associated credibility interval (95% HPD). The scale at the bottom of the figure is reported in millions of years before the present.

**Figure 4:**
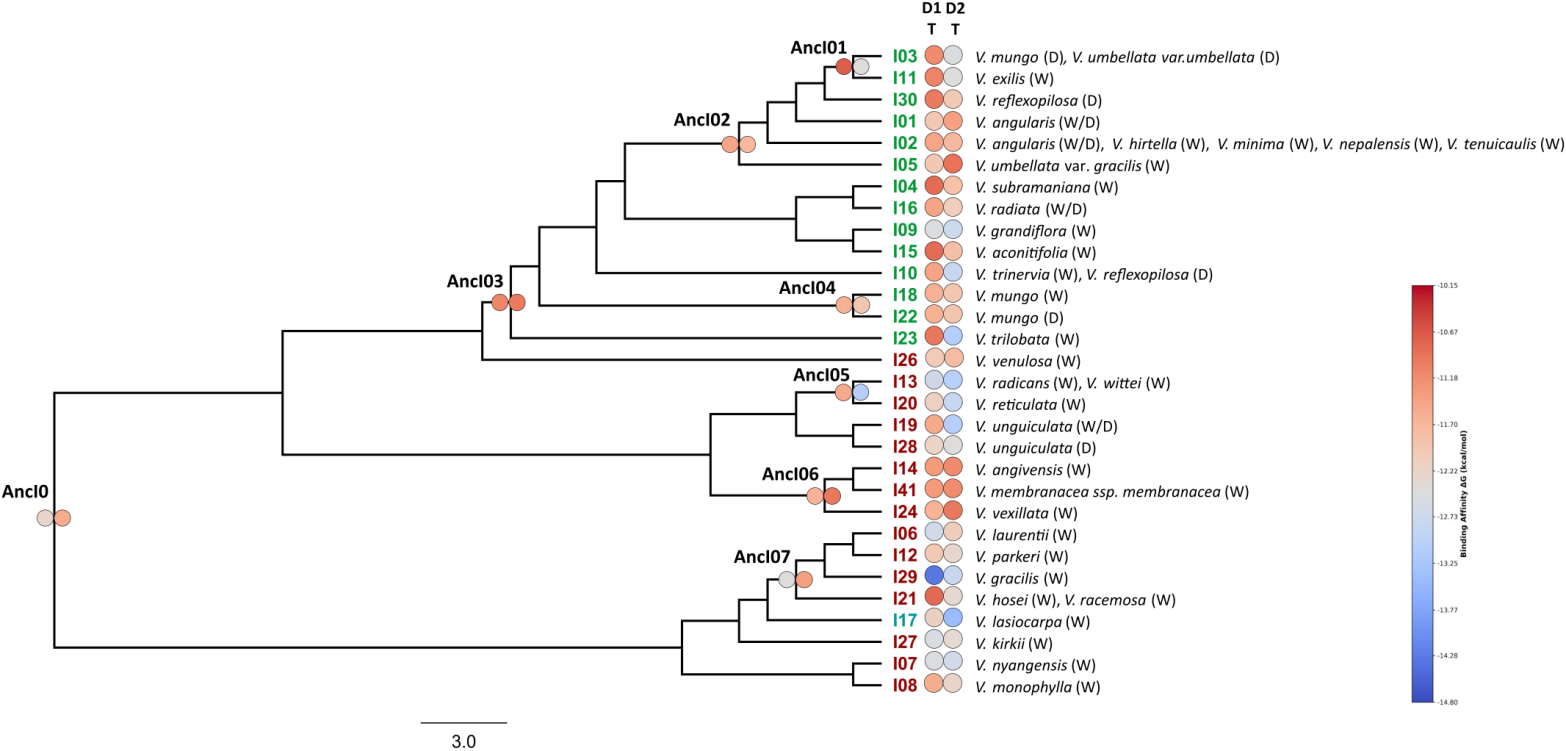
The ML tree, produced by IQ-TREE and based on the *BBI1* (BBI genes part of the *BBI1* orthogroup) alignment, displays the isoform codes on the tips (green = Asia, red = Africa, azure = America) and the ancient isoforms inferred by GRASP on the nodes. For each highly supported node (SH-aLRT>90/Approximate Bayes Test>0.95/Ultrafast bootstrap>90) are indicated the ancient isoform codes. The circles are colored according to the level of interaction energy between domains (D1 = first domain and D2 = second domain) and target (T = trypsin). The taxa are classified as wild (W) and domesticated (D).

**Figure 5:**
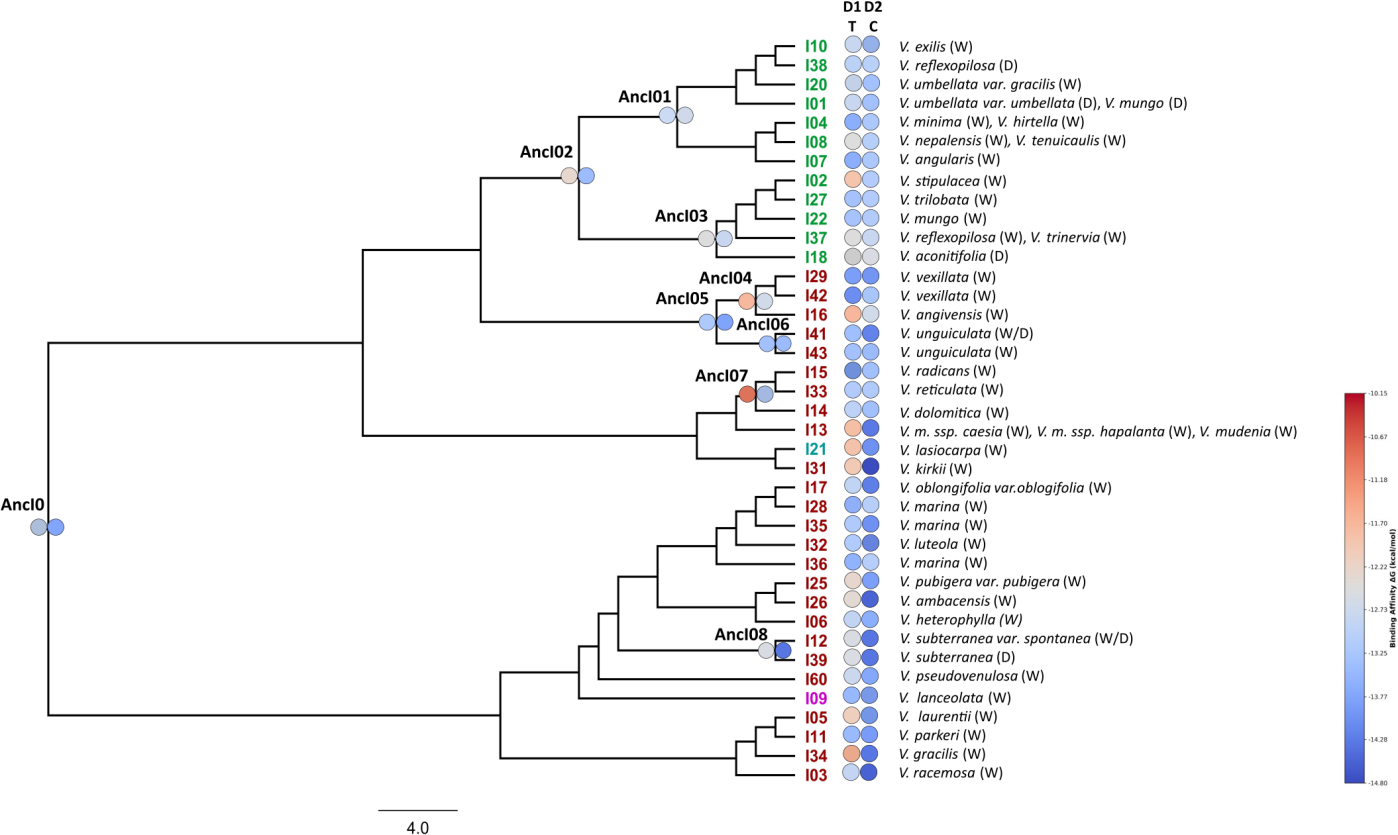
The ML tree, produced by IQ-TREE and based on the *BBI2* (BBI genes part of the *BBI2* orthogroup) alignment, displays the isoform codes on the tips (green = Asia, red = Africa, azure = America) and the ancient isoforms inferred by GRASP on the nodes. For each highly supported node (SH-aLRT>90/Approximate Bayes Test>0.95/Ultrafast bootstrap>90) are indicated the ancient isoform codes. The circles are colored according to the level of interaction energy between domains (D1 = first domain and D2 = second domain) and targets (T = trypsin; C = chymotrypsin). The taxa are classified as wild (W) and domesticated (D). V.m. = *Vigna membranacea*.

A total of 134 sequences were produced in this work and the sequences obtained were deposited in the NCBI GenBank database (PV010864-PV010940). Two multiple sequence alignments of 76 DNA sequences for *BBI1* and 83 DNA sequences for *BBI2* were produced for successive analysis (**Supplementary Tables S8**, **S9**, **S10** and **S11**). The phylogenetic trees produced by two independent MCMC runs were combined to obtain a maximum clade credibility tree. The Bayesian estimates of divergence times for each gene are presented in **Figures 2**, **3**. For both maximum clade credibility trees, the most ancient lineages originated in Africa. Moreover, the stem of monophyletic Asian clade is estimated to have diverged from other *Vigna* species about 6.5 million years ago for *BBI1* and 7.5 million years ago for *BBI2*. Site Model test showed that both genes are targeted by forces of positive selection during evolution. The Likelihood Ratio Tests were significant, indicating that a fraction of sites is evolving under strong adaptive pressure: 2LnL = 10.3047, p < 0.005 (5.9%, ω=2.76) for BBI1 and 2LnL = 29.9319, p < 0.001 (10.6%, ω=3.52) for BBI2. In particular, the reactive residue of the first domain that determines the interaction with the trypsin (P1) is evolving under positive selection for all analyses performed. In P1 we found the following amino acids: Arginine, Lysine and Alanine for *BBI1* and Arginine, Lysine, Histidine and Glutamic Acid for *BBI2*. The complete list of residues under positive selection, identified by FUBAR, MEME, FEB and BEB analysis, are summarized in **Supplementary Table S12**. Phylogenetic relationships between isoforms are shown in **Figures 4**, **5**. On each tip and on each highly supported node (SH-aLRT>90/Approximate Bayes Test>0.95/Ultrafast bootstrap>90) are reported the interacting energy values with the targets (ML-trees in newick format are shown in **Supplementary Table S13**). Finally, ancient amino acid sequences (AncI) are reconstructed by inferring phylogenetic relationships between modern isoforms (I) and the list of modern and ancient amino acid sequences is reported in **Supplementary Material** (**Supplementary Figures S5**, **S6**).

### 3.4 Interaction energy with the main targets

**Figure 6:**
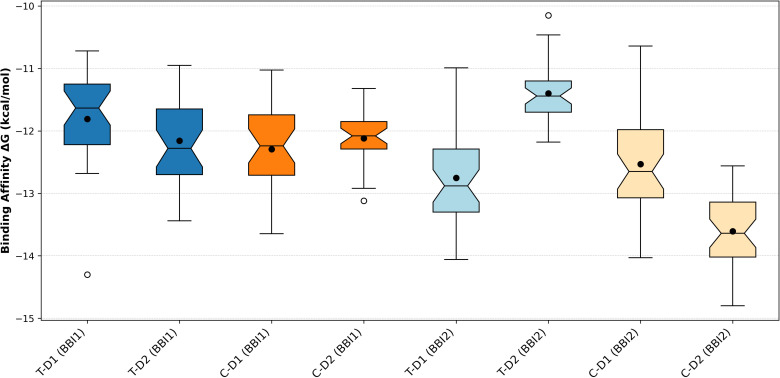
Box-plot representing the distribution values of ΔGbinding calculated for each system. BBI1= BBI genes part of the *BBI1* orthogroup; BBI2= BBI genes part of the *BBI2* orthogroup; D1 = first domain; D2 = second domain; T = trypsin target; C = chymotrypsin target. The box extremes represent values between 25 and 75%, while the solid line represents the median, and the whiskers represent values between 5 and 95% of the data distribution. The black circles represent the average values whereas the white circles the outlier values.

Molecular docking simulations were performed for all BBI variants to assess their affinity for human trypsin and chymotrypsin. The cumulative ΔGbinding values for trypsin and chymotrypsin calculated for the BBI1 are reported in the box plot graph in blue and orange (**Figure 6**). These data show that the domain 1 (D1) binds chymotrypsin slightly better than trypsin. In contrast, the D2 region exhibits identical affinity for both trypsin and chymotrypsin. Taking into account the individual DeltaG values for the D1 region (**Supplementary Figure S5**), the P31 variant (corresponds to isoform I29 in **Figure 4**) shows the highest inhibitory activity, with a strong affinity to trypsin (more than -14 kcal/mol), whereas most other BBIs prefer to bind chymotrypsin. The D2 region shows no clear specificity for trypsin or chymotrypsin (**Supplementary Figure S6**). Moreover, our results suggest an inverse relationship: when a variant has a stronger binding affinity for trypsin, it tends to have a weaker binding affinity for chymotrypsin, and vice versa.

The cumulative ΔGbinding values for trypsin and chymotrypsin calculated for the BBI2 are reported in light blue and yellow in the box plot graph. The D1 region has the same affinity to both trypsin and chymotrypsin (**Supplementary Figure S6**). In contrast, the D2 region shows a stronger affinity to chymotrypsin than to trypsin (**Supplementary Figure S7**). While most variants in the D1 region have similar binding affinities for both trypsin and chymotrypsin, the P42, P34, P23, and P14 variants (respectively I42, I29, I15, I4 in **Figure 5**) have a significantly stronger affinity for trypsin.

An overall analysis of the BBI1 and BBI2 data indicates that BBI2 generally shows higher affinity for both trypsin and chymotrypsin compared to BBI1, except for pocket D2, which shows lower average affinity values specifically for trypsin.

## 4 Discussion

BBIs are an important family of serine protease inhibitors, and their high rate of duplication events combined with the forces of natural selection drive the formation of divergent expression profiles and the development of new functional roles (Xie et al., 2021). Our results revealed a common expression pattern across all the seeds analyzed, evidencing that *BBI1* and *BBI2* genes have the highest expression level when compared with other BBI genes. This difference was confirmed in all species studied (**Figure 1**). The *BBI1* and *BBI2* genes correspond to the two genes located on chromosome 4 in the *Vigna unguiculata* (cowpea) reference genome available in the NCBI database^6^. In this species, their predicted inhibitory targets are trypsin–trypsin and trypsin–chymotrypsin, respectively. However, due to the uncertainty regarding target specificity across different species, in this work they are referred to as BBI1 and BBI2.

The high level of concentration of these two genes expressed in seeds, combined with their ability to inhibit different serine proteases, enhances defenses during germination and seedling growth, protecting the plant from potential pathogen attacks during its most vulnerable developmental stage. However, some authors have also interpreted that the abundance of serine protease inhibitors in seeds participate in the regulation of endogenous protease activity and serve as sulfur storage proteins because of their particularly high cysteine content (Pusztai, 1972; Tsybina et al., 2001). Several protease families are required in seeds and storage organs for protein turnover and their activity is regulated by endogenous inhibitors (Vorster et al., 2023). Therefore, the abundant concentration of BBIs in seeds might serve a triple role. Beyond providing protection against non-plant organisms, they could also regulate endogenous proteases during the mobilization of reserve proteins. Our results also revealed that the expression levels of BBIs in leaves are highly variable. This result is consistent with their ability to exhibit differential expression during various life cycles of the plant or in response to different environmental conditions (Eckelkamp et al., 1993; Malefo et al., 2020). Due to the involvement of BBIs in responses to abiotic and biotic stress, the sensitivity of BBI gene expression in leaves to environmental variations could have significant implications for agriculture (Dramé et al., 2013; Chen et al., 2024). However, additional experiments are required to evaluate the gene expression levels of BBIs across various developmental stages and environmental conditions, as well as to comprehend how to optimize the production of these inhibitors. It is also important to acknowledge the limitations of relying on a single reference gene. Although actin is commonly used, its stability may fluctuate under different conditions (Ruan and Lai, 2007). Therefore, incorporating multiple candidate reference genes is preferable for identifying the most stable one under the experimental conditions, especially in detailed studies examining gene expression across various stages and environments.

To better understand the variability and functionality of *BBI1* and *BBI2* genes, we have analysed two datasets of DNA sequences including their homologs identified in 42 *Vigna* species. A phylogenetic analysis demonstrated that the amino acid residue P1 of the first domain, which is expected to confer inhibitory specificity for trypsin, is under positive selection in both genes. The interaction with protease is primarily determined by the P1 residue, and alterations to this residue significantly influence the inhibitor’s specificity (Clemente and Domoney, 2006). In general, positive selection promotes genetic variants that increase the organism’s fitness, favouring changes in amino acid residues to optimize adaptation to environmental pressures. At the P1 residue of the first domain, in addition to the amino acids arginine and lysine which commonly target trypsin, we identified alanine (in BBI1) which emerged in some African species, as well as histidine and glutamic acid (in BBI2) found in *V. racemosa* and *V. stipulacea*, respectively. According to the literature, when alanine is at P1, the BBI is known to inhibit elastase, a protease associated with various medical conditions, including inflammatory processes and their corresponding immune responses (Kawabata et al., 2002; Polverino et al., 2017; Ferreira et al., 2019). Evidence of elastase inhibitory activity, potentially linked to the BBI family, has also been recently reported in cowpea and it was suggested that natural inhibitors targeting elastase could hold potential for medical trials and therapeutic applications (Ferreira et al., 2019). The interaction between alanine and elastase has been widely predicted in the literature and confirmed in studies on other species (Clemente and Domoney, 2006; Rocco et al., 2011; De Paola et al., 2012). Meanwhile, the molecular targets of the other two amino acids, glutamic acid and histidine, remain largely unknown and have been much less investigated. While some studies described glutamic acid as inactive in this context (Clemente and Domoney, 2006), we hypothesize that histidine exhibits affinity for trypsin due to its positive charge. Therefore, based on all these findings, we propose that the P1 residue of the first domain has undergone continuous changes throughout evolution and that the residues defining the interaction have attempted to optimize their affinity with different proteases. As confirmation of this, BBI1 and BBI2 sequenced from different species exhibited varying energy requirements, but interaction with chymotrypsin in the second domain of BBI2 proved to be generally favored (**Figures 4**–**6**). The fact that this latter interaction generally requires less binding energy is attributed to the presence of amino acid residues at the P1 position which confer a higher specificity toward chymotrypsin. However, two important aspects must be considered for a correct interpretation of our results. Firstly, amino acids variability observed in the P1 residue seem insufficient to fully explain varying energy requirements. Additional residues, particularly those within the binding loops, play a significant role in determining inhibitory specificity (**Supplementary Figures S5**, **S6**, **S7** and **S8**). Unfortunately, our current findings do not permit quantification of their individual contributions. Therefore, more in-depth analyses are needed to precisely characterize these interactions and the specific roles of these residues. Secondly, the interaction values obtained in this study should be interpreted as a preference of a specific domain for a particular target. We cannot rule out the possibility that, in nature, the domain may also bind to targets other than the one defined by P1.

Finally, observations of affinity values along the phylogenetic trees did not reveal any evolutionary pattern within the *Vigna* genus. Although our sampling was primarily concentrated in Africa and Asia, the ability of BBI1 and BBI2 to interact with the proteases analyzed in this study does not appear to be correlated with particular phylogenetic groups. Given the relatively recent origin of the *Vigna* genus (likely about ten million years ago), this timeframe may have been too short to create lineages with particularly favored interaction activity. However, the modern protein variants observed across different species have exhibited a wide range of interaction values, with differences that are not negligible. The inclusion of many taxa in the sampling allowed to increase the capability of exploration and new protein variants were found. The wide availability of wild *Vigna* species is an important resource for African and Asian countries and the findings obtained may prove useful for breeding programs or genome editing efforts aimed at selecting more efficient cultivars. However, to fully exploit the potential of BBIs for the benefit of both agriculture and human health, a nuanced approach is needed, that takes into account the differential specificity and the application of advanced breeding and genetic engineering techniques to tailor BBI profiles. Indeed, the complex and seemingly paradoxical nature of BBIs, which act as antinutrients by inhibiting digestive proteases in humans and pests, but also possess health-promoting properties, poses a difficult challenge. Nevertheless, there is now a range of strategies that can be deployed to address this issue. For example, precision genetic editing technology, such as CRISPR/Cas9, and RNA interference (RNAi) technology has been successfully applied to soybeans, resulting in a substantial reduction in trypsin and chymotrypsin inhibitory activity and improving digestibility without adversely affecting other nutritional characteristics (Kim et al., 2024, 2025). Similar strategies could also be applied in *Vigna*. In order to enhance specific human health benefits, it is necessary to identify or genetically manipulate BBI variants that interact more strongly with disease-related human proteases, but which bind less strongly to human digestive proteases. The “double-headed” structure of BBIs, prevalent in legumes like *Vigna*, with two distinct inhibitory domains that simultaneously and independently inhibit two different protease molecules provides a powerful platform for engineering. Using site-directed mutagenesis, one inhibitory site could be modified to reduce its binding to human digest enzymes and the other site (or a different BBI variant) could be modified to enhance its binding to specific human proteases involved in disease pathways. Concurrently, conventional breeding programmes, augmented by molecular markers and advanced phenotyping, could identify existing germplasm for BBI variants that inherently exhibit a more favourable balance between lower antinutritional activity for humans and enhanced activity against specific insect proteases and/or proteases that are beneficial to humans.

In this context, our findings offer a useful starting point for addressing this challenge. We highlight that docking simulations provide a valuable preliminary assessment of protein-target interaction potential, though biological validation is still required. Docking simulations applied to new genes encoding specific proteins represent an inexpensive and efficient method for the preliminary evaluation of protein variants hidden in wild and domesticated accessions.

## 5 Conclusion

Our findings on the high expression of *BBI1* and *BBI2* genes in *Vigna* seeds align with previous hypotheses suggesting a triple role in plant defense, regulation of endogenous protease activity, and storage protein function. We acknowledge that our expression data, derived from a representative subset of species and potentially influenced by non-specificity of some primer, provides only a preliminary view; confirming these patterns across a broader phylogenetic spectrum is an essential next step. Despite these considerations, our comprehensive evolutionary analysis across 42 species identifies the P1 residue of the first domain under positive selection. However, we interpret this selection signature with caution, recognizing that non-adaptive forces, such as genetic drift during population bottlenecks from distinct domestication events on different continents, can also elevate dN/dS ratios, thus creating signatures that mimic positive selection. This selective pressure, whatever its origin, appears to have driven a dynamic functional diversification. Our exploratory *in silico* docking, which provided an initial assessment of this diversity, was based on human protease models. Future studies must therefore validate these interactions using ecologically relevant targets, such as modeled insect or fungal enzymes, to truly understand their co-evolutionary context. Ultimately, this rich reservoir of natural variation presents a valuable genetic toolkit for future agricultural applications. We therefore present our conclusions as a robust working hypothesis, where the compelling genetic and initial computational insights now require large-scale transcriptomic and direct biological validation to fully harness their potential.

## Data Availability

The datasets presented in this study can be found in online repositories. The names of the repository/repositories and accession number(s) can be found below: https://www.ncbi.nlm.nih.gov/, GenBank locus from PV010864 to PV010940.

## Footnotes

1 http://merops.sanger.ac.uk.

2 https://www.ebi.ac.uk/merops/cgi-bin/famsum?family=I12.

3 http://beast.bio.ed.ac.uk/Tracer.

4 http://tree.bio.ed.ac.uk/software/figtree.

5 https://github.com/haddocking/haddock3.

6 https://www.ncbi.nlm.nih.gov/datasets/genome/GCF_004118075.2/.
