# Supplementary material for: Gene expression and evolution of Bowman-Birk protease inhibitors in wild and domesticated Vigna (Fabaceae) species
Source: Front Plant Sci. 2026 Jan 28;16:1657741. doi: 10.3389/fpls.2025.1657741 (PMC12894363; doi:10.3389/fpls.2025.1657741)
Supplement: Supplementary file 1 [file Table1.docx]

Gene expression and evolution of Bowman-Birk protease inhibitors (BBIs) in wild and domesticated *Vigna* (Fabaceae) species

Supplementary Material

**The following Supporting Information is available for this article:**

[1 Supplementary Figures 3](#_Toc205811501)

[Supplementary Figure 1. 3](#_Toc205811502)

[Supplementary Figure 2. 3](#_Toc205811503)

[Supplementary Figure 3. 4](#_Toc205811504)

[Supplementary Figure 4. 5](#_Toc205811505)

[Supplementary Figure 5. 6](#_Toc205811506)

[Supplementary Figure 6. 8](#_Toc205811507)

[Supplementary Figure 7. 10](#_Toc205811508)

[Supplementary Figure 8. 12](#_Toc205811509)

[2 Appendix 14](#_Toc205811510)

[RNA extraction 14](#_Toc205811511)

[Real Time PCR protocol 14](#_Toc205811512)

[3 Supplementary Tables 15](#_Toc205811513)

[Supplementary Tables 1. 15](#_Toc205811514)

[Supplementary Tables 2. 16](#_Toc205811515)

[Supplementary Tables 3. 17](#_Toc205811516)

[Supplementary Tables 4. 20](#_Toc205811517)

[Supplementary Tables 5. 23](#_Toc205811518)

[Supplementary Tables 6. 24](#_Toc205811519)

[Supplementary Tables 7. 26](#_Toc205811520)

[Supplementary Tables 8. 27](#_Toc205811521)

[Supplementary Tables 9. 32](#_Toc205811522)

[Supplementary Tables 10. 36](#_Toc205811523)

[Supplementary Tables 11. 37](#_Toc205811524)

[Supplementary Tables 12. 38](#_Toc205811525)

[Supplementary Tables 13. 38](#_Toc205811526)

[4 References 40](#_Toc205811527)

# Supplementary Figures

Supplementary Figure 1. Graphical representations of amino acid sequences for *BBI1* and *BBI2* identified in *V. unguiculata*. Portions of the amino acid sequences are coloured differently: signal peptide in blue, propeptide in red and mature protein in green. The two interactive domains are underlined, and the amino acid residues, involved in the primary contact that confers inhibitory specificity, are represented in bold (trypsin/trypsin for BBI1 and trypsin/chymotrypsin for BBI2).


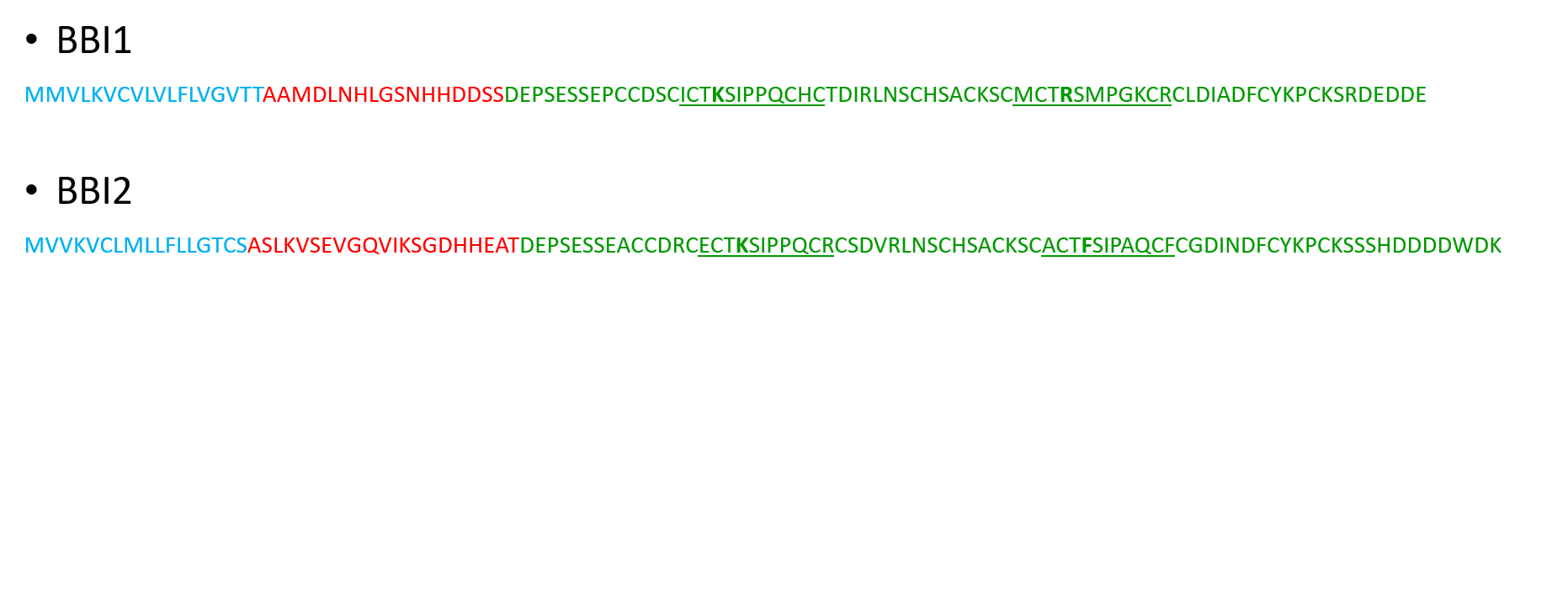


Supplementary Figure 2. Photographical collection of seeds of each accession. Photo was taken using a Nikon D3500 equipped with a Nikon 18-140 mm lens.


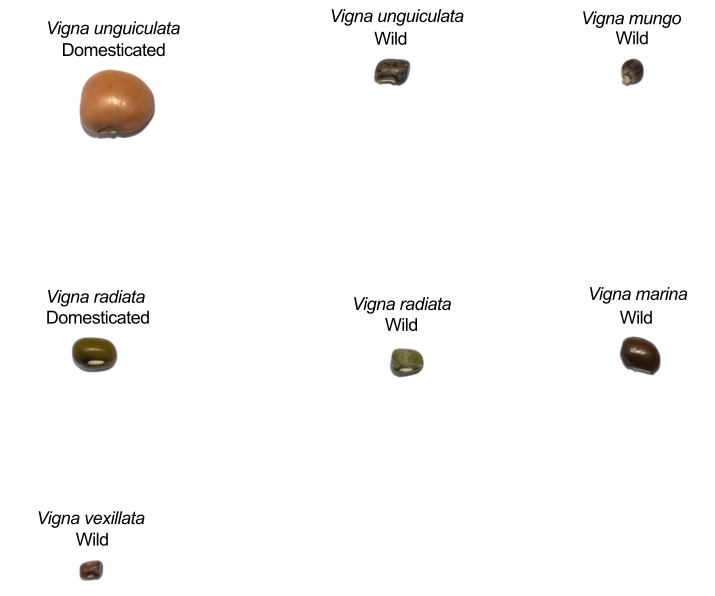


Supplementary Figure 3. Consensus tree of BBI genes identified in the 12 *Vigna* genomes analysed. The tree was visualized and rooted at midpoint using FigTree 1.4.4 (http://tree.bio.ed.ac.uk/software/figtree). In the tree, the clades of the different orthogroups resulting from the OrthoFinder 2.5.5 (Emms and Kelly, 2019) are highlighted using different colours. Each gene from a different orthogroup is followed by a number, and multiple genes from the same species within an orthogroup are followed by a letter.

**
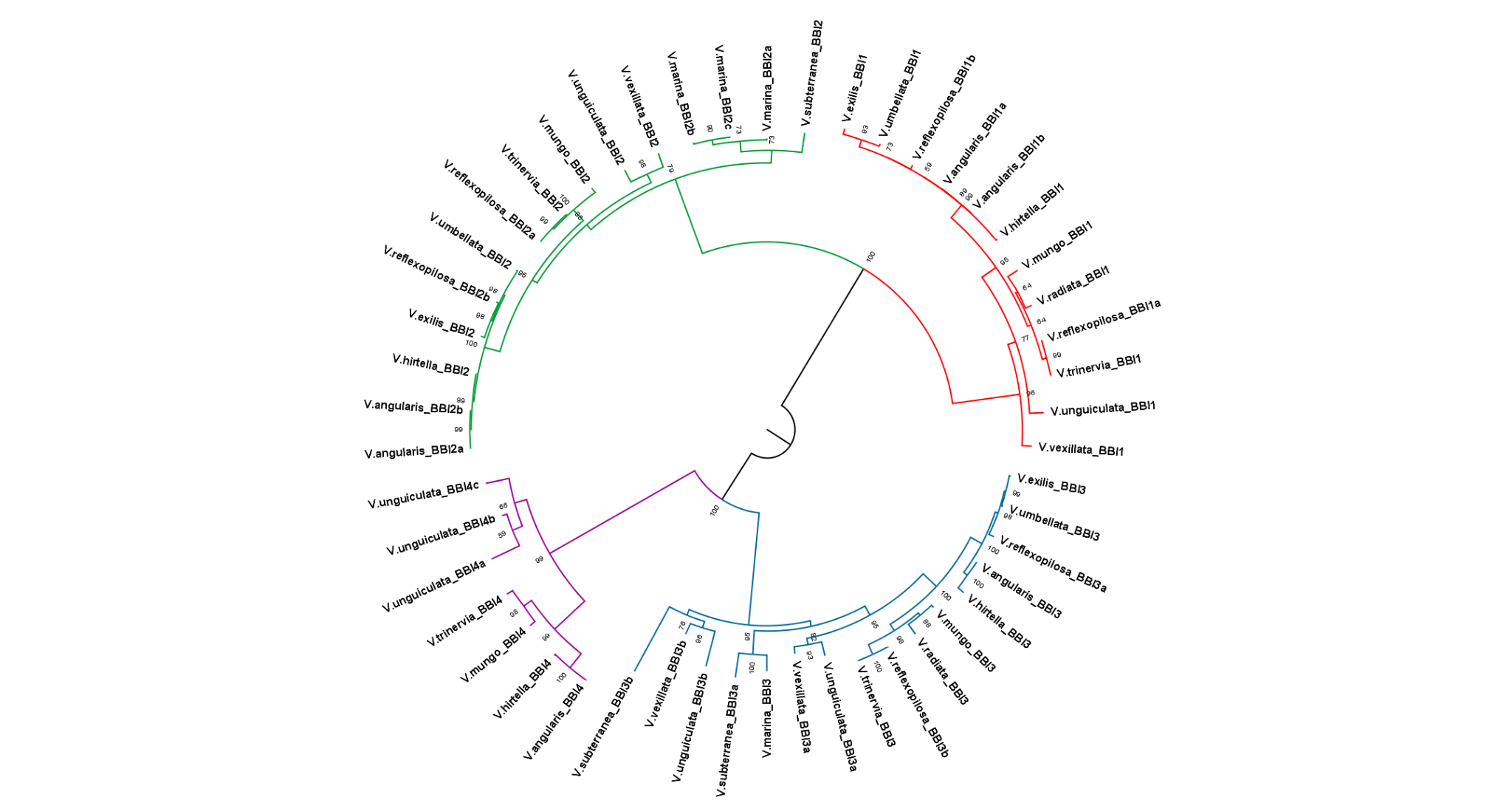
**

Supplementary Figure 4. The figure shows the plDDT values for each predicted structure. The orange area indicates plDDT < 50 (very low confident), the light blue area indicates 50 < plDDT < 70 (low confident), the cyan area indicates 70 < plDDT < 90 (good confident), and the dark blue area indicates plDDT > 90 (high confident).

**
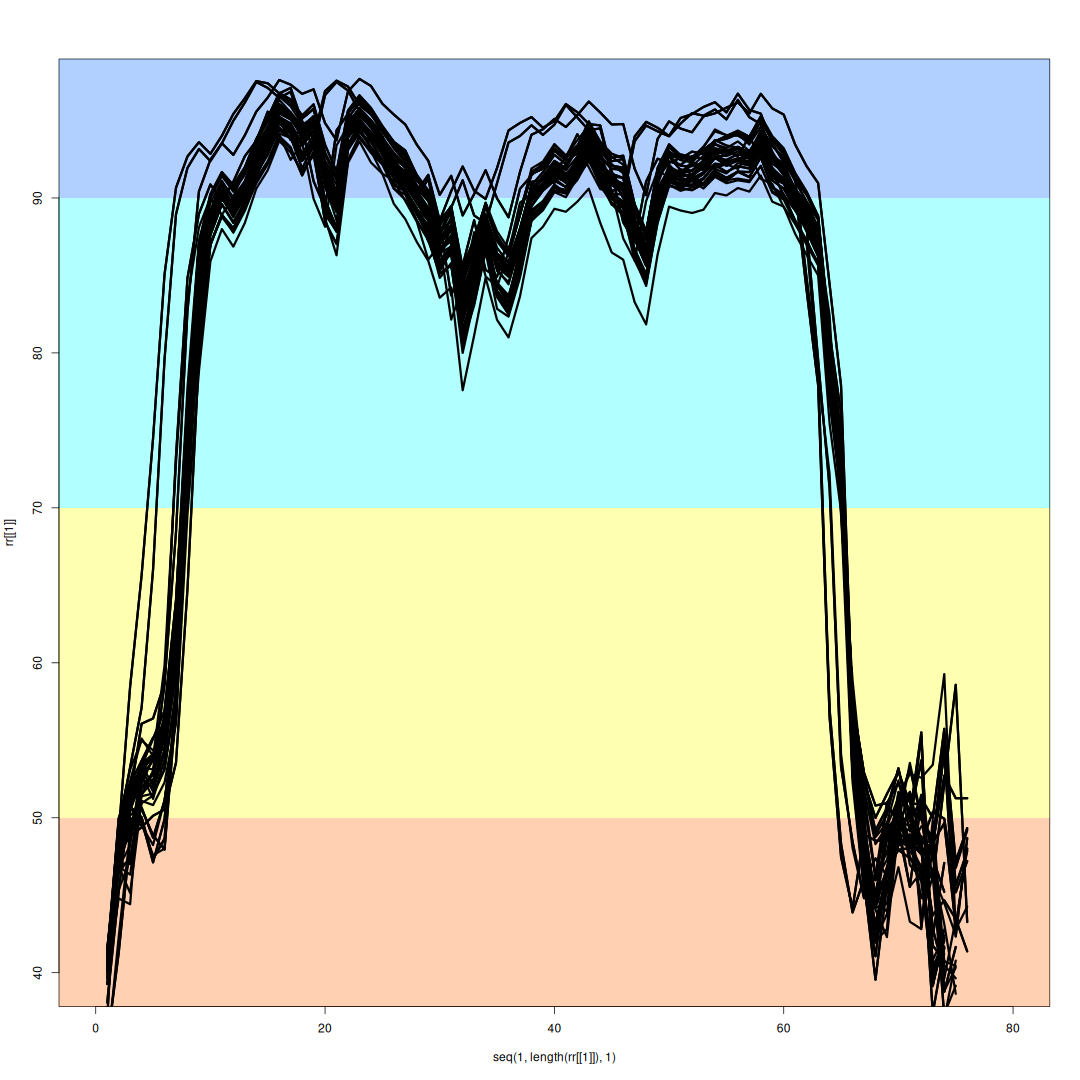
**

Supplementary Figure 5. a) Bar chart of ΔG_binding_ of Trypsyn (blue) and Chymotrypsin (orange) to the domain 1 (D1) of BBI1. The t-test was used to determine if the difference in binding affinities between trypsin and chymotrypsin was significant or simply due to chance for each variant. The error bar is the standard deviation obtained from ΔG_binding_ values of all the poses of the best scored cluster. Grey bar shows a significant difference between trypsin and chymotrypsin. b and c) Sequence alignment of BBI1 variants. Bold letters highlight the residues that differ in each variant from the one with the lowest affinity value with the interacting protein, while colored boxes show the amino acids interacting (whitin 5 Å) with trypsin (b) or chymotrypsin (c). The color scale represents the ΔG_binding_ values (from lowest affinity in red to highest affinity in blue).

**
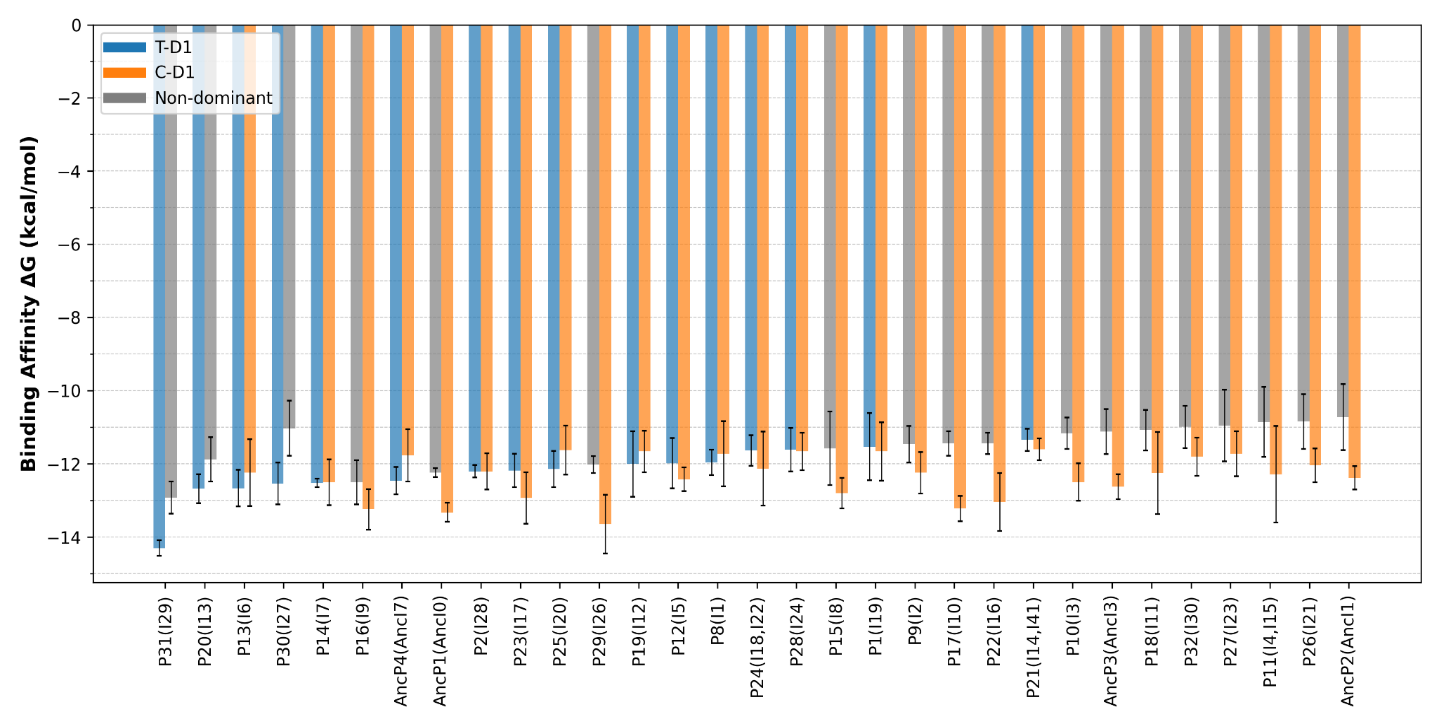
a)**

**
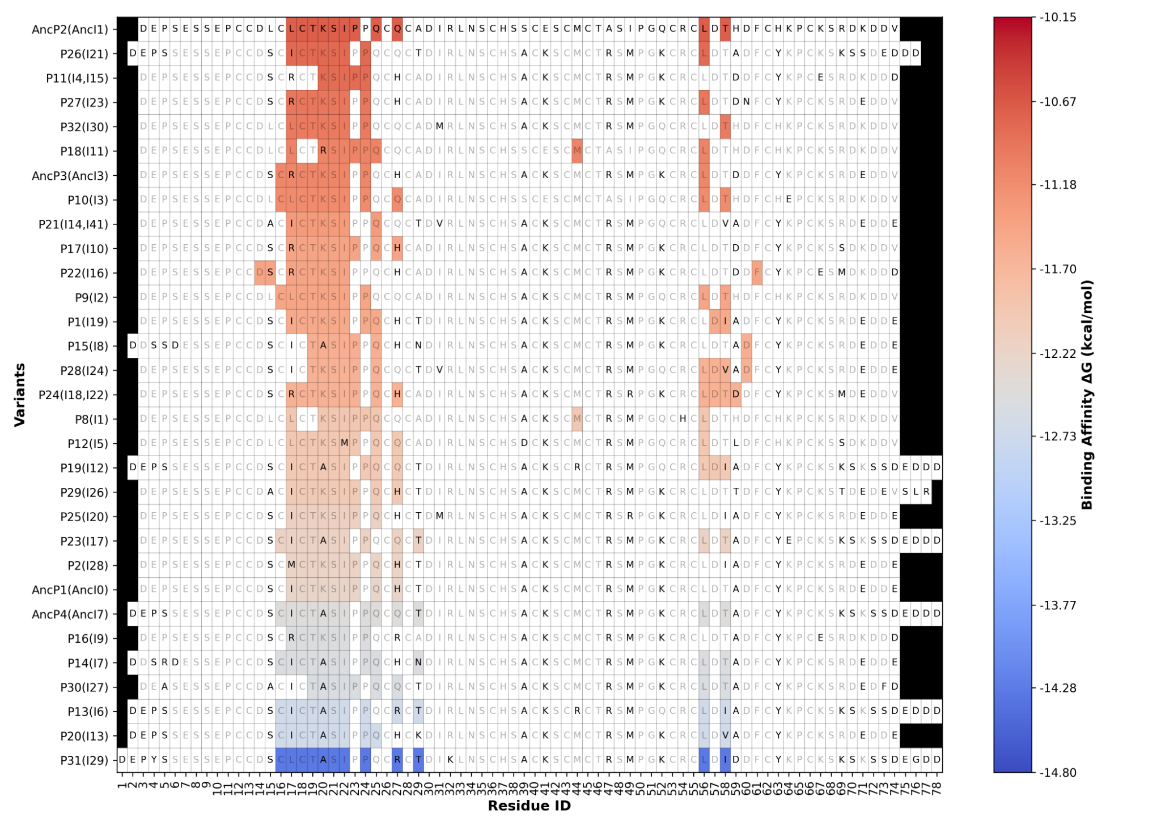
b)**

**
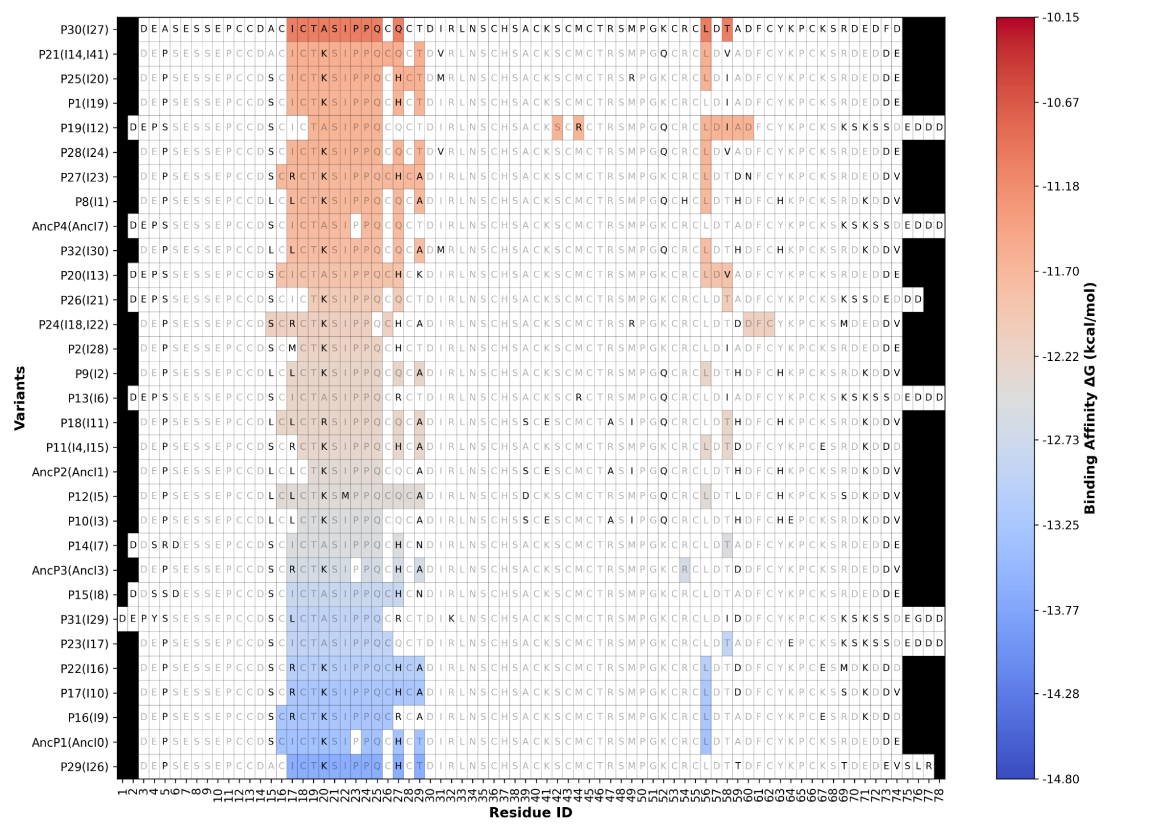
c)**

Supplementary Figure 6. Bar chart of ΔGbinding of Trypsyn (blue) and Chymotrypsin (orange) to the domain 2 (D2) of BBI1. The t-test was used to determine if the difference in binding affinities between trypsin and chymotrypsin was significant or simply due to chance for each variant. The error bar is the standard deviation obtained from ΔGbinding values of all the poses of the best scored cluster. Grey bar shows a significant difference between trypsin and chymotrypsin. b and c) Sequence alignment of BBI1 variants. Bold letters highlight the residues that differ in each variant from the one with the lowest affinity value with the interacting protein, while colored boxes show the amino acids interacting (whitin 5 Å) with trypsin (b) or chymotrypsin (c). The color scale represents the ΔGbinding values (from lowest affinity in red to highest affinity in blue).

**a)**

**
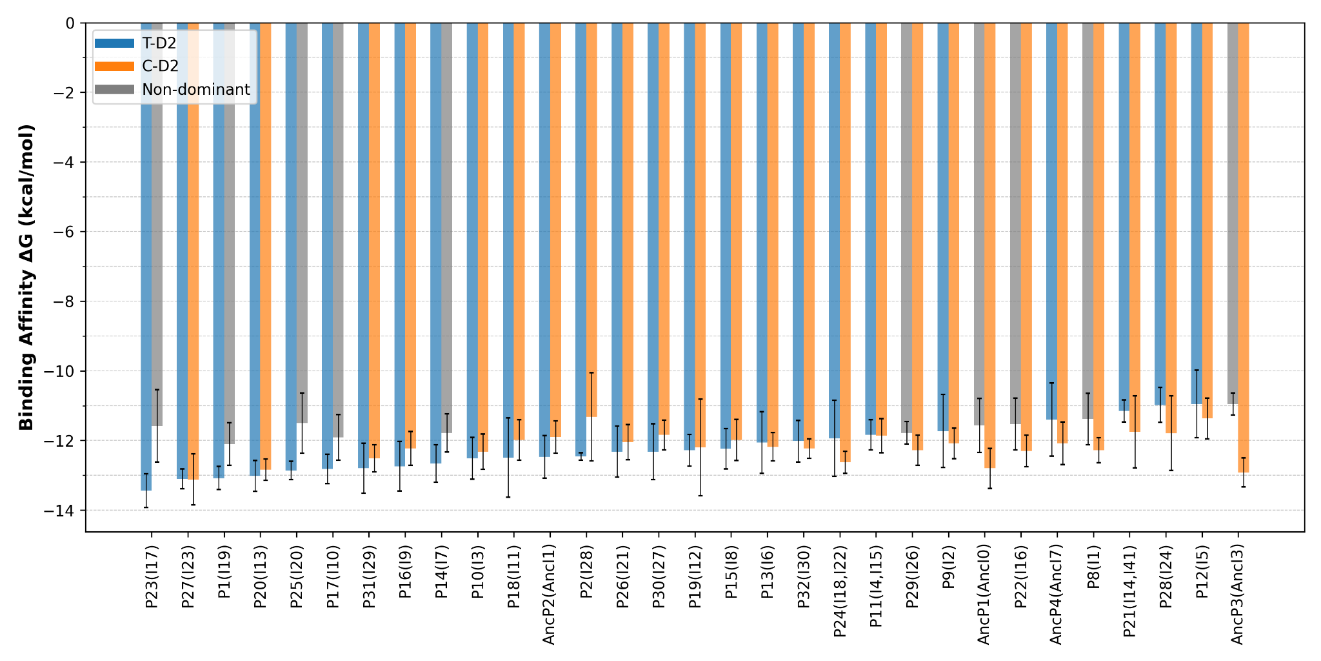
**

**
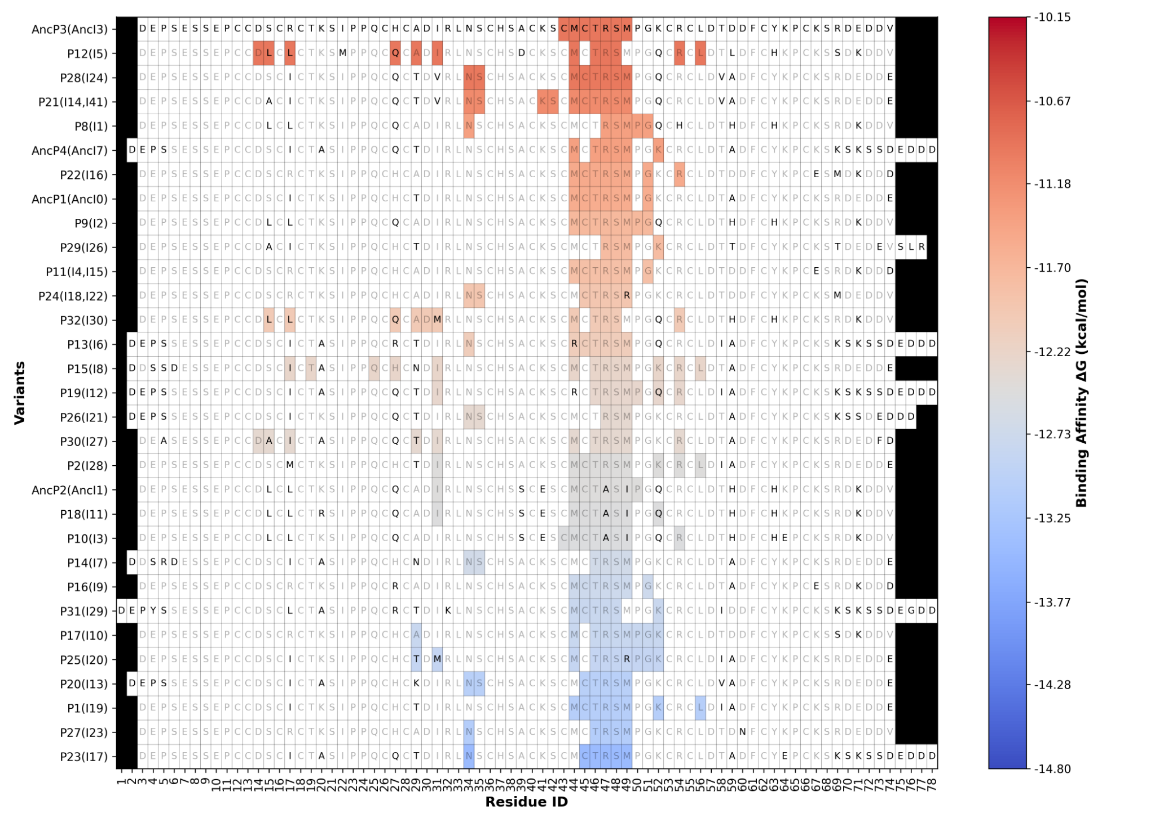
b)**

**c)**

**
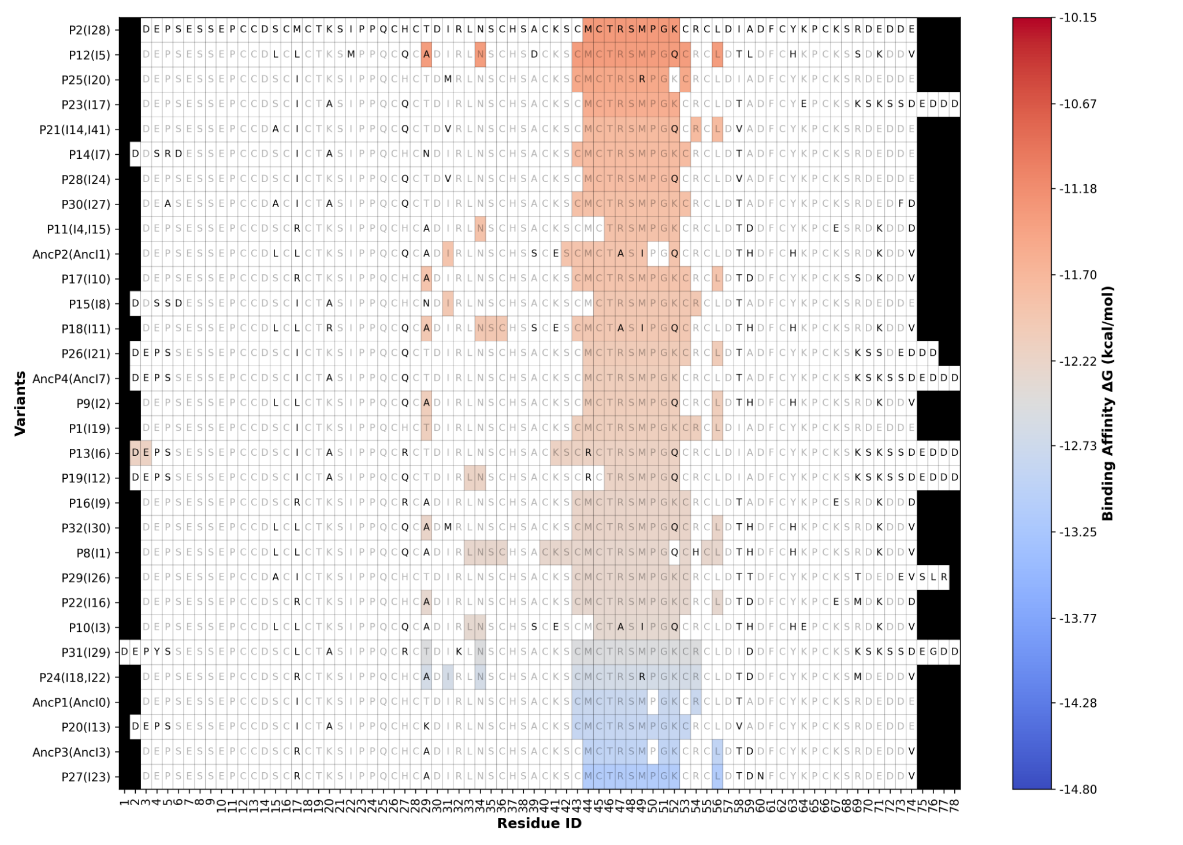
**

Supplementary Figure 7. Bar chart of ΔGbinding of Trypsin (blue) and Chymotrypsin (orange) to the domain 1 (D1) of BBI2. The t-test was used to determine if the difference in binding affinities between trypsin and chymotrypsin was significant or simply due to chance for each variant. The error bar is the standard deviation obtained from ΔGbinding values of all the poses of the best scored cluster. Grey bar shows a significant difference between trypsin and chymotrypsin. b and c) Sequence alignment of BBI2 variants. Bold letters highlight the residues that differ in each variant from the one with the lowest affinity value with the interacting protein, while colored boxes show the amino acids interacting (whitin 5 Å) with trypsin (b) or chymotrypsin (c). The color scale represents the ΔGbinding values (from lowest affinity in red to highest affinity in blue).


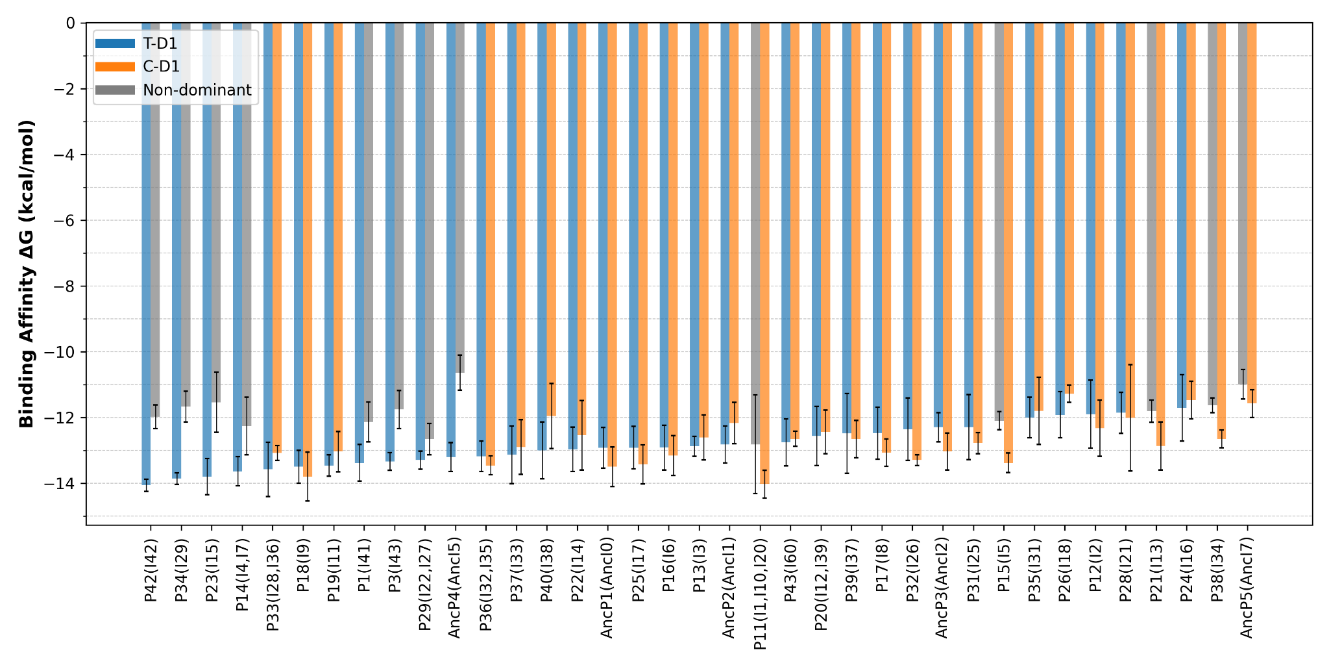
**a)**


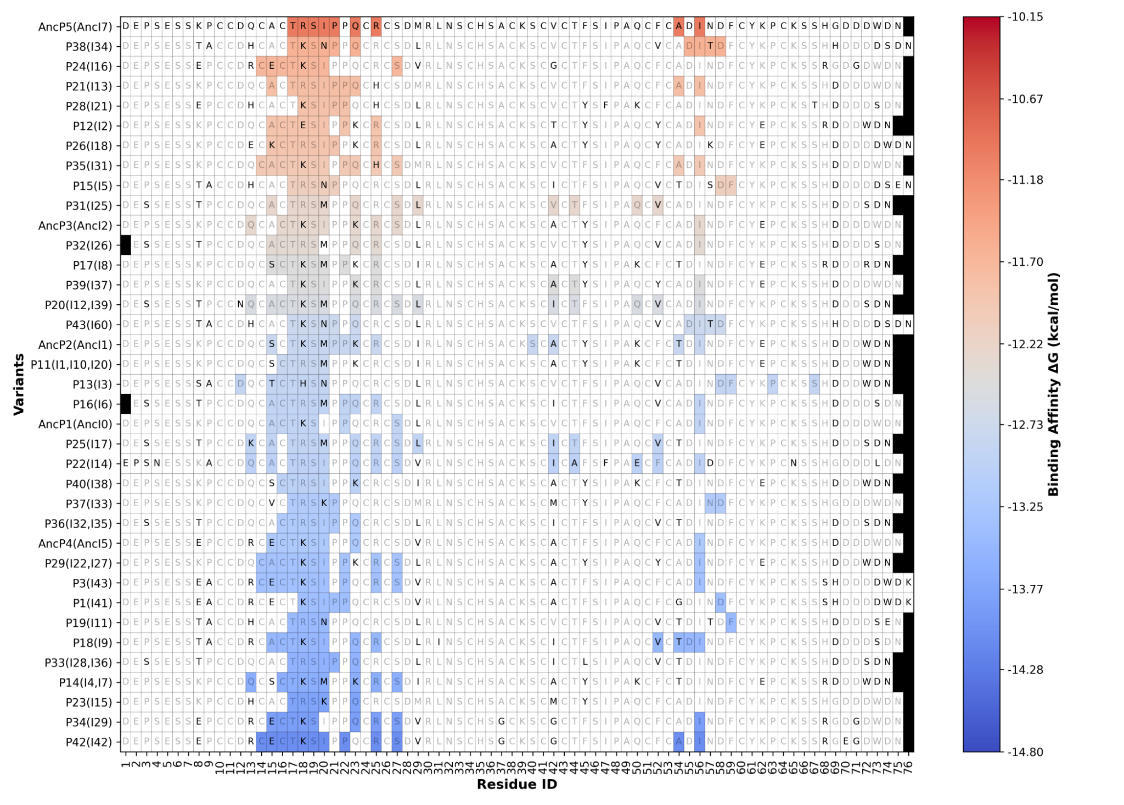
**b)**


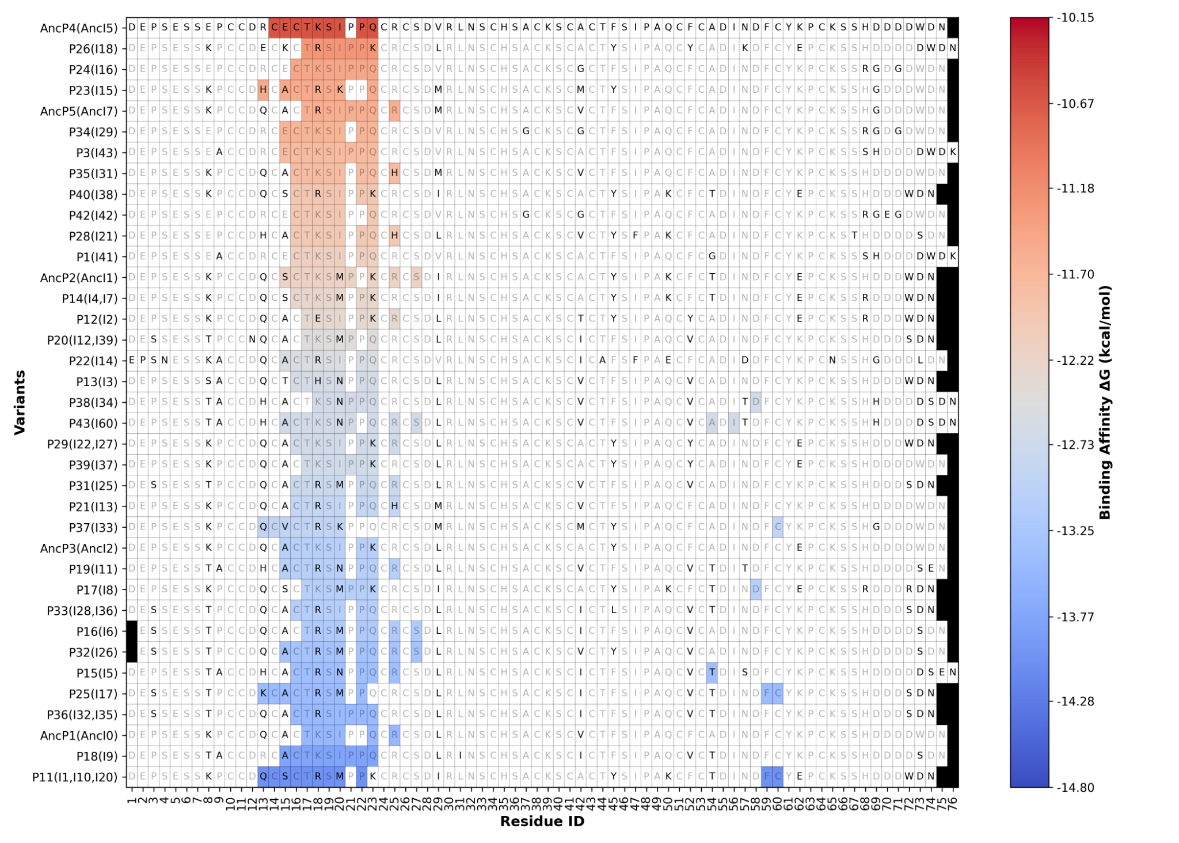
**c)**

Supplementary Figure 8. Bar chart of ΔGbinding of Trypsyn (blue) and Chymotrypsin (orange) to the domain 2 (D2) of BBI2. The t-test was used to determine if the difference in binding affinities between trypsin and chymotrypsin was significant or simply due to chance for each variant. The error bar is the standard deviation obtained from ΔGbinding values of all the poses of the best scored cluster. Grey bar shows a significant difference between trypsin and chymotrypsin. b and c) Sequence alignment of BBI2 variants. Bold letters highlight the residues that differ in each variant from the one with the lowest affinity value with the interacting protein, while colored boxes show the amino acids interacting (whitin 5 Å) with trypsin (b) or chymotrypsin (c). The color scale represents the ΔGbinding values (from lowest affinity in red to highest affinity in blue).


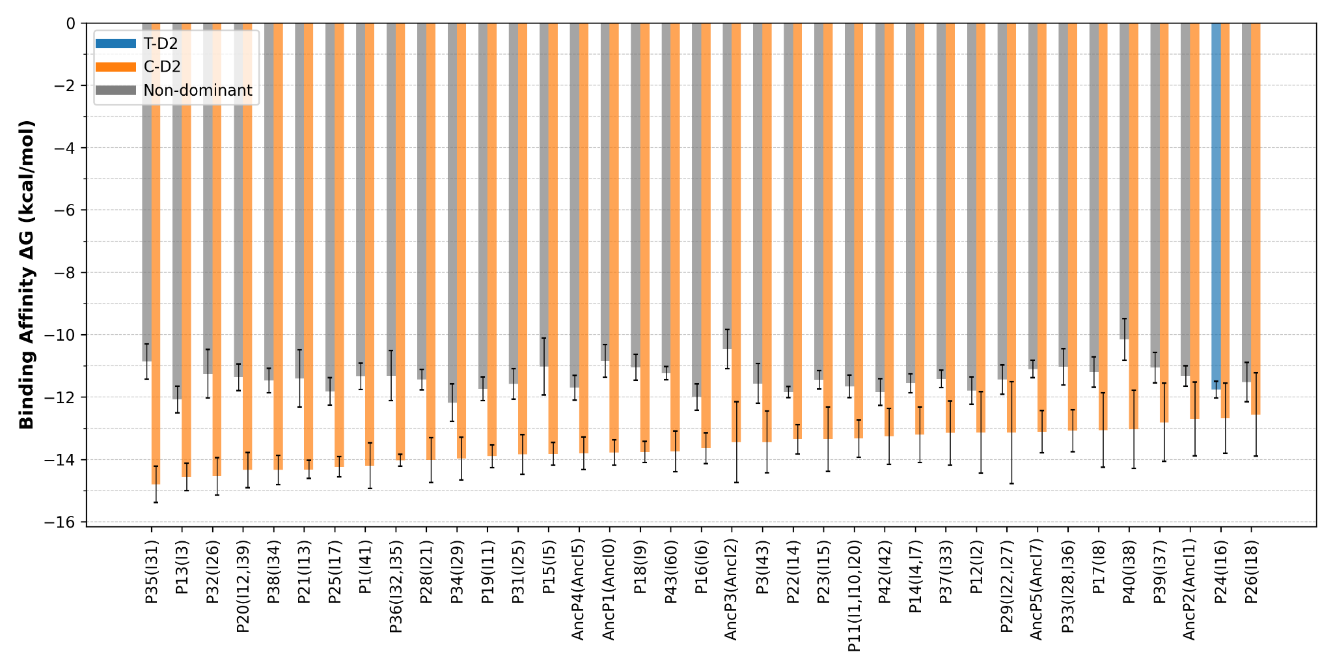
**a)**

**
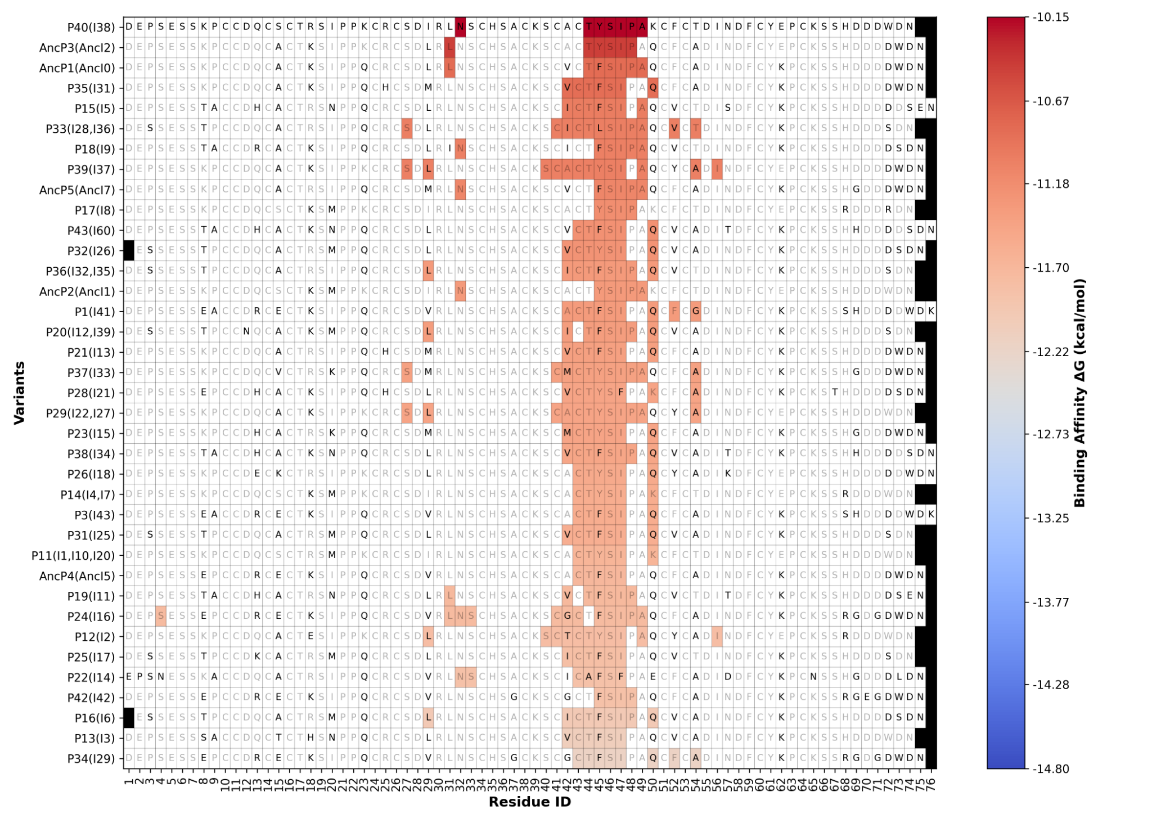
b)**

**
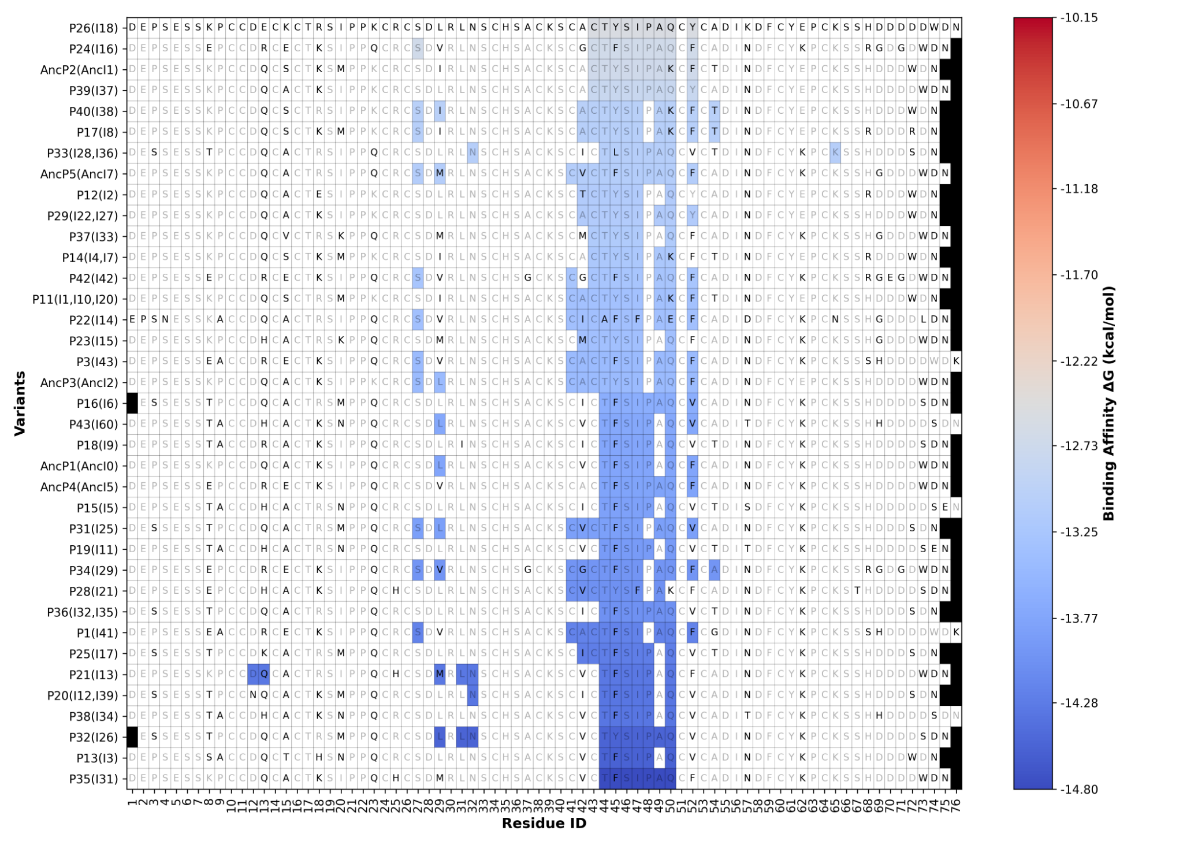
c)**

# Appendix

RNA extraction: The leaves were frozen in liquid nitrogen and grinded to a fine powder by using a Mini Bead Beater (Biospec Products) with zirconia/silica beads (2.5 mm). The seeds were washed, immediately frozen with liquid nitrogen and then grinded by mortar and pestle, while kept frozen with liquid nitrogen. Then, 450 μL of RLC buffer with DTT was added and vortex-mixed. The lysate was transferred to a QIAshredder column and centrifuged at full speed. A 0.5 volume of EtOH (>96%) was added to the supernatant mixing by pipetting. The sample was loaded into an RNeasy Mini spin column and centrifuged. 700 μL of buffer RW1 were loaded, and centrifuged. 500 μl of buffer RPE were loaded and centrifuged, for two times. A centrifuge then was performed to dry the column and, at last, the RNA was eluted with sterile 40 μl MilliQ water.

Real Time PCR protocol: The PCR reaction was set as suggested by the producer, briefly: holding stage at 95°C for 60 seconds, cycling stage for 45 times with denaturation at 95°C for 15 seconds and extension at 60°C for 30 seconds, melt curve stage at 95° for 15 seconds and 60°C for 60 seconds and then a +0.3°C ramp up until 95°C with plate read. Every sample was replicated at least 3 times, and for each mix at least 3 blanks were made. To design primer pairs, different software were used to guarantee the best primer combinations possible: primer3web v4.1.0 (Untergasser et al., 2012) and NCBI Primer-Blast (https://www.ncbi.nlm.nih.gov/tools/primer-blast/). A final manual control and editing step was then performed using BioEdit v. 7.2.5 (Hall, 1999). Primer pairs were optimized to have a similar melting temperature, a GC content between 40% and 70% and to amplify between 100-200 bp.

**PCR Products control**

For each primer pair used in the Real-Time PCR experiment, PCR products were analyzed by electrophoresis on a 2% agarose gel for approximately 30 minutes. PCR reactions were carried out with the following mix: 15 ul gotaq green mastermix (Promega), 1 ul forward primer, 1 ul reverse primer, 15 ng of cDNA (1.5 ul of the 10 ng/ul operative cDNA solution) and 11.5 ul Nuclease Free Water. The reaction protocol was the following: initial denaturation, 95°C for 5 minutes, 40 cycles made of denaturation, 95°C for 30 seconds, annealing, 56°C for 30 seconds, extension, 72°C for 30 seconds, final elongation at 72°C for 2 minutes. PCR products were run in a 2% agarose electrophoresis gel for about 30 minutes.

# Supplementary Tables

Supplementary Tables 1. List of genomes used in this study. In the table are reported the name of species, the code of identification (ID), the name of the database (DB) used and the link.

| **Species** | **ID** | **DB** | **Link** |
| --- | --- | --- | --- |
| *V. angularis* | GCA016808095_1 | NCBI | https://www.ncbi.nlm.nih.gov/datasets/genome/GCF_016808095.1/ |
| *V. hirtella* | GCA027742795_1 | NCBI | https://www.ncbi.nlm.nih.gov/datasets/genome/GCA_027742795.1/ |
| *V. mungo* | GCA019096145_1 | NCBI | https://www.ncbi.nlm.nih.gov/datasets/genome/GCA_019096145.1/ |
| *V. reflexopilosa* | GCA027742785_1 | NCBI | https://www.ncbi.nlm.nih.gov/datasets/genome/GCA_027742785.1/ |
| *V. trinervia* | GCA027743565_1 | NCBI | https://www.ncbi.nlm.nih.gov/datasets/genome/GCA_027743565.1/ |
| *V. umbellata* | GCA025174585_1 | NCBI | https://www.ncbi.nlm.nih.gov/datasets/genome/GCA_025174585.1/ |
| *V. unguiculata* | GCA004118075.2 | NCBI | https://www.ncbi.nlm.nih.gov/datasets/genome/GCF_004118075.2/ |
| *V. radiata* | GCA000741045_2 | NCBI | https://www.ncbi.nlm.nih.gov/datasets/genome/GCF_000741045.1/ |
| *V. exilis* | Vexilis v1 | VIGGS | https://viggs.dna.affrc.go.jp/viewer/gbrowse/exilis_v1a1/ |
| *V. marina* | Vmarina v1 | VIGGS | https://viggs.dna.affrc.go.jp/viewer/gbrowse/marina_v1a1/ |
| *V. vexillata* | Vvaxillata v1 | VIGGS | https://viggs.dna.affrc.go.jp/viewer/gbrowse/vexillata_v1a1/ |
| *V. subterranea* | Vigsu genome | Orcae | https://bioinformatics.psb.ugent.be/orcae/aocc/overview/Vigsu |

Supplementary Tables 2. List of selected accessions for RNA expression. ID = Code of identification. Form was classified as W = wild and D = domesticated. Accessions with TVNU and TVU codes were obtained from International Institute of Tropical Agriculture (https://my.iita.org/accession2/, IITA, Ibadan, Nigeria). *Vigna radiat*a domesticated (RC) was obtained from Mashan Shanshan Agricultural Technology Ltd., Mashan County, China.

| **ID** | **Species** | **Form** | **Origin** |
| --- | --- | --- | --- |
| TVNU 1174 | *V. marina* | W | Gabon |
| TVNU 1076 | *V. mungo* | W | India |
| TVNU 1250 | *V. radiata* | W | Ghana |
| TVNU 969 | *V. vexillata* | W | Zimbabwe |
| TVNU 1506 | *V. unguiculata* ssp. *dekindtiana* | W | Togo |
| TVU 15207 | *V. unguiculata* ssp. *unguiculata* | D | Congo |
| RC | *V. radiata* | D | China |

Supplementary Tables 3. List of selected accessions to explore the gene variability of *BBI1* and *BBI2* genes. For each accession the identification code, taxonomical classification and provenience of seeds are reported. Form indicates plants wild (W) or domesticated (D) and N indicates the number of individuals analysed. Accessions with TVNU and TVU codes were obtained from International Institute of Tropical Agriculture (https://my.iita.org/accession2/, IITA, Ibadan, Nigeria), those with NI code from “Meise Botanic Garden” (https://www.plantentuinmeise.be/en/) and finally with BGE code from “Centro Nacional de Recursos Fitogeneticos” (CNRF, https://www.inia.es/Pages/Home.aspx). While *V. radiat*a domesticated (RC) was obtained from Mashan Shanshan Agricultural Technology Ltd., Mashan County, China.

| **Accession** | **Species** | **Subspecies** | **Variety/ Cultivar** | **Provenience** | **Form** | **N** |
| --- | --- | --- | --- | --- | --- | --- |
| NI482 | *V.aconitifolia* |  |  | India | D | 2 |
| TVNU1150 | *V.ambacensis* |  |  | Central African Republic | W | 2 |
| NI378 | *V. angivensis* |  |  | Madagascar | W | 2 |
| NI1634 | *V. angularis* |  |  | Japan | W | 2 |
| BGE022149 | *V.angularis* |  |  | Madrid | D | 2 |
| NI2203 | *V.dolomitica* |  |  | Congo | W | 1 |
| NI1926 | *V.exilis* |  |  | Thailand | W | 2 |
| TVNU610 | *V. gracilis* |  |  | Congo | W | 2 |
| NI1721 | *V.grandiflora* |  |  | Thailand | W | 1 |
| NI1485 | *V.heterophylla* |  |  | Cameroon | W | 2 |
| NI1377 | *V. hirtella* |  |  | Thailand | W | 2 |
| TVNU1147 | *V. hosei* |  |  | Botswana | W | 2 |
| TVNU22 | *V. kirkii* |  |  | Zaire | W | 2 |
| TVNU364 | *V. kirkii* |  |  | Malawi | W | 2 |
| NI1860 | *V.lanceolata* |  |  | Australia | W | 1 |
| TVNU1018 | *V.lasiocarpa* |  |  | Costa Rica | W | 3 |
| NI1434 | *V.laurentii* |  |  | Cameroon | W | 2 |
| TVNU487 | *V. luteola* |  |  | Kenya | W | 1 |
| TVNU1441 | *V. marina* |  |  | Mozambique | W | 1 |
| TVNU890 | *V.membranacea* | *caesia* |  | Kenya | W | 1 |
| TVNU897 | *V.membranacea* | *caesia* |  | Kenya | W | 1 |
| TVNU146 | *V.membranacea* | *hapalantha* |  | Ghana | W | 2 |
| NI1448 | *V.membranacea* | *membranacea* |  | Ethiopia | W | 3 |
| NI1376 | *V. minima* |  |  | Thailand | W | 2 |
| NI1564 | *V.monophylla* |  |  | Zimbabwe | W | 2 |
| NI2152 | *V.mudenia* |  |  | South Africa | W | 2 |
| TVNU1076 | *V. mungo* |  |  | India | W | 2 |
| TVNU1268 | *V. mungo* |  |  | Japan | W | 1 |
| BGE040528 | *V.mungo* |  |  | Madrid | D | 2 |
| NI1704 | *V.nepalensis* |  |  | Nepal | W | 2 |
| NI1522 | *V.nyangensis* |  |  | Zimbabwe | W | 1 |
| NI461 | *V.oblongifolia* |  | *oblongifolia* | Congo | W | 2 |
| NI1969 | *V. parkeri* |  |  | Kenya | W | 2 |
| NI410 | *V. pseudovenulosa* |  |  | Senegal | W | 1 |
| NI1147 | *V.pubigera* |  | *pubigera* | Ghana | W | 1 |
| TVNU973 | *V.racemosa* |  |  | Mali | W | 2 |
| TVNU1521 | *V.racemosa* |  |  | Benin | W | 1 |
| TVNU1250 | *V. radiata* |  |  | Ghana | W | 2 |
| RC | *V. radiata* |  |  | China | D | 2 |
| NI238 | *V.radicans* |  |  | Congo | W | 1 |
| TVNU1104 | *V.reticulata* |  |  | Malawi | W | 3 |
| NI1030 | *V.stipulacea* |  |  | Guyana | D | 1 |
| NI1135 | *V.subramaniana* |  |  | India | W | 2 |
| TVSU1295 | *V.subterranea* |  |  | Central african republic | D | 1 |
| TVSU305 | *V.subterranea* |  |  | Burkina Faso | D | 1 |
| NI2008 | *V.subterranea* |  | *spontanea* | Central African Republic | W | 2 |
| NI1927 | *V.tenuicaulis* |  |  | Thailand | W | 2 |
| TVNU1332 | *V.trilobata* |  |  | India | W | 2 |
| NI1737 | *V.trinervia* |  |  | Thailand | W | 2 |
| NI1398 | *V.umbellata* |  | *gracilis* | Thailand | W | 3 |
| NI21 | *V.umbellata* |  | *umbellata* | Rwanda | D | 2 |
| TVNU179 | *V.venulosa* |  |  | Nigeria | W | 1 |
| TVNU1476 | *V. vexillata* |  |  | Swaziland | W | 2 |
| TVNU332 | *V. wittei* |  |  | Zambia | W | 2 |

Supplementary Tables 4. List of primers used in real time PCR analysis for each species. Two pair of primers have amplified more than one gene (*V. unguiculata* *BBI4a-BBI4b-BBI4c*, *V. marina* *BBI2a-BBI2b-BBI2c*).

| **Species** | **Target gene** | **Sense** | **DNA sequence** |
| --- | --- | --- | --- |
| *V. unguiculata* | *BBI2* | F | AGTGCTAGCTTGAAGGTGTCT |
| *V. unguiculata* | *BBI2* | R | GTCTGAACAGCGGCATTGAG |
| *V. unguiculata* | *BBI1* | F | GGGGTTACTACTGCAGCCAT |
| *V. unguiculata* | *BBI1* | R | CAAGCCGAGTGACACGAATT |
| *V. unguiculata* | *BBI3a* | F | CCTAGGGTTCTCTGCGACAA |
| *V. unguiculata* | *BBI3a* | R | GGAGGAATTGAGCGCGTG |
| *V. unguiculata* | *BBI3b* | F | TCCTCCACAGTGTCATTGCA |
| *V. unguiculata* | *BBI3b* | R | GGTTTGGCATCTTCATCTGAGT |
| *V. unguiculata* | *BBI4a-BBI4b-BBI4c* | F | TGATAGTTTTCAGTGCAGTGGA |
| *V. unguiculata* | *BBI4a-BBI4b-BBI4c* | R | CATCGGCATTGTGGAGGAAT |
| *V. marina* | *BBI3* | F | TCGATTCGATGCAAGTTCCT |
| *V. marina* | *BBI3* | R | GAGTTTGGGTTCATCAGAGTGG |
| *V. marina* | *BBI2a-BBI2b-BBI2c* | F | ACCATGCTGTGATCAATGCG |
| *V. marina* | *BBI2a-BBI2b-BBI2c* | R | TCATCGTGACTGGACTTGCA |
| *V. mungo* | *BBI2* | F | TCTGGGAACTTGTGCTGCTA |
| *V. mungo* | *BBI2* | R | GAACAGCGGCATTTAGGAGG |
| *V. mungo* | *BBI1* | F | TGGTGCTAAAGGTGTGTGTG |
| *V. mungo* | *BBI1* | R | TACAGGATTTGCAAGCGGAG |
| *V. mungo* | *BBI3* | F | TTTTGCTCCTAGGGTTCGCC |
| *V. mungo* | *BBI3* | R | AGGAGTCACAGCACGCTTTC |
| *V. mungo* | *BBI4* | F | CTATTGCTGTGGATGCTCGC |
| *V. mungo* | *BBI4* | R | CATGCAACGACACTGAGGAG |
| *V. radiata* | *BBI1* | F | GGGGTTACTACTGCTGGCAT |
| *V. radiata* | *BBI1* | R | GCCTGGCATTGATCGTGTAC |
| *V. radiata* | *BBI3* | F | TCGCTTCGATCCAACATCCT |
| *V. radiata* | *BBI3* | R | CGGTTCATCTGAGTGACATGG |
| *V. vexillata* | *BBI2* | F | GCTAGGCCAACTCATCAAAAGT |
| *V. vexillata* | *BBI2* | R | GAAGTCGTTTATGTCCGCACAA |
| *V. vexillata* | *BBI1* | F | ATGAATCTGAACCACCTCAGAAG |
| *V. vexillata* | *BBI1* | R | CAGCAACATCAAGACAACGACA |
| *V. vexillata* | *BBI3a* | F | GTTGCCCATGCACAAAATCACT |
| *V. vexillata* | *BBI3a* | R | TGTTTGCCCATTATGAGTGAGC |
| *V. vexillata* | *BBI3b* | F | CTGTACATGCACAAGTTCAATTCC |
| *V. vexillata* | *BBI3* | R | TACTGAGTTATGTTTGCCATTATGA |
| All species | Actin | F | GCGTGACCTCACTGATGCC |
| All species | Actin | R | TCGCAATCCACATCTGTTGG |

Supplementary Tables 5. List of primers used to amplify and sequence the *BBI1* gene.

| **Sequences** | **Accessions** |
| --- | --- |
| **Forward:** ACATGCAGAAAGCAACAAA  **Reverse:** TGTCTTCCCTTACACAACAA | V.aconitifolia_NI482_1, V.aconitifolia_NI482_2, V.angivensis_NI378_2, V.angivensis_NI378_3, V.angularis_BGE022149_1, V.angularis_BGE022149_2, V.angularis_NI1634_2, V.angularis_NI1634_3, V.exilis_NI1926_1, V.exilis_NI1926_2, V.grandiflora_NI1721_1, V.hirtella_NI1377_1, V.hirtella_NI1377_2, V.hosei_TVNU1147_1, V.hosei_TVNU1147_2, V.kirkii_TVNU22_2, V.lasiocarpa_TVNU1018_2, V.lasiocarpa_TVNU1018_3, V.laurentii_NI1434_1, V.membranacea_ssp.membranacea_NI1448_1, V.membranacea_ssp.membranacea_NI1448_2, V.membranacea_ssp.membranacea_NI1448_3, V.minima_NI1376_1, V.minima_NI1376_2, V.monophylla_NI1564_1, V.monophylla_NI1564_2, V.mungo_BGE040528_1, V.mungo_BGE040528_2, V.mungo_TVNU1076_2, V.mungo_TVNU1268_2, V.nepalensis_NI1704_1, V.nepalensis_NI1704_2, V.nyangensis_NI1522_1, V.parkeri_NI1969_1, V.parkeri_NI1969_2, V.racemosa_TVNU973_2, V.racemosa_TVNU973_3, V.racemosa_TVNU1521_3, V.radiata_RC1, V.radiata_RC2, V.radiata_TVNU1250_1, V.radiata_TVNU1250_2, V.radicans_NI238_1, V.reticulata_TVNU1104_2, V.reticulata_TVNU1104_3, V.subramaniana_NI1135_1, V.subramaniana_NI1135_2, V.tenuicaulis_NI1927_1, V.trilobata_TVNU1332_1, V.trilobata_TVNU1332_2, V.trinervia_NI1737_3, V.umbellata_var.umbellata_NI21_1, V.umbellata_NI21_2 |
| **Forward:** CAATGCAGAAAGCAACAAA  **Reverse:** TGTCTTCCCTTACACAACAA | V.tenuicaulis_NI1927_2, V.umbellata_var.gracilis_NI1398_1, V.umbellata_var.gracilis_NI1398_2, V.umbellata_var.gracilis_NI1398_3 |

Supplementary Tables 6. List of primers were used to amplify and to sequence the *BBI2* gene.

| **Sequences** | **Accessions** |
| --- | --- |
| **Forward:** AGATGGGTTTGAACAACAAC  **Reverse:** GAAAGGGCTTCATCCATTT | V.heterophylla_NI1485_1, V.heterophylla_NI1485_2, V.kirkii_TVNU22_2, V.kirkii_TVNU22_3, V.kirkii_TVNU364_2, V.kirkii_TVNU364_3, V.lanceolata_NI1860_1, V.lanceolata_NI1860_2, V.lasiocarpa_TVNU1018_1, V.lasiocarpa_TVNU1018_2, V.lasiocarpa_TVNU1018_3, V.laurentii_NI1434_2, V.marina_NI1441_1, V.pubigera_var.pubigera_NI1147_2, V.racemosa_TVNU973_2, V.racemosa_TVNU973_3, V.stipulacea_NI1030_1, V.subterranea_TVSU305_1, V.subterranea_TVSU_1295_1, V.subterranea_var.spontanea_NI2008_1, V.subterranea_var.spontanea_NI2008_2 |
| **Forward:** CCATGCAGAAAACAACGAAC  **Reverse:** GAAAGGGCTTCATCCATTT | V.aconitifolia_NI482_1, V.ambacensis_TVNU1150_1, V.ambacensis_TVNU1150_2, V.angivensis_NI378_2, V.angivensis_NI378_3, V.angularis_NI1634_2, V.angularis_NI1634_3, V.dolomitica_NI2203_1, V.exilis_NI1926_1, V.exilis_NI1926_2, V.gracilis_TVNU610_1, V.hirtella_NI1377_1, V.hirtella_NI1377_2, V.minima_NI1376_2, V.mudenia_NI2152_1, V.mudenia_NI2152_2, V.mungo_BGE040528_1, V.mungo_BGE040528_2, V.mungo_TVNU1076_1, V.mungo_TVNU1076_2, V.mungo_TVNU1268_2, V.nepalensis_NI1704_1, V.nepalensis_NI1704_2, V.radicans_NI238_1, V.reticulata_TVNU1104_1, V.reticulata_TVNU1104_2, V.stipulacea_NI1030_2, V.tenuicaulis_NI1927_1, V.trilobata_TVNU1332_1, V.trilobata_TVNU1332_2, V.umbellata_NI21_1, V.umbellata_var.umbellata_NI21_2, V.umbellata_var.gracilis_NI1398_1, V.umbellata_var.gracilis_NI1398_2 |
| **Forward:** GAAAACAACGAAGACATCAC  **Reverse:** GAAAGGGCTTCATCCATTT | V.gracilis_TVNU610_2, V.laurentii_NI1434_1, V.oblongifolia_var.oblongifolia_NI461_1, V.oblongifolia_var.oblongifolia_NI461_2, V.parkeri_NI1969_1, V.parkeri_NI1969_2, V.pseudovenulosa_NI410_2, V.vexillata_TVNU1476_1, V.vexillata_TVNU1476_2 |
| **Forward:** GAGAGCAAGAACAACACAATGG  **Reverse:** CCGAAAGGGCTTCATCCATTTT | V.membranacea_ssp.hapalantha_TVNU146_1, V.membranacea_ssp.hapalantha_TVNU146_2, V.minima_NI1376_1 |

Supplementary Tables 7. Comparison of gene expression levels in seeds and leaves through T-test analysis.

| **Specie and genes** | **average Ct seed** | **average Ct leaf** | **p value** |
| --- | --- | --- | --- |
| V. marina BBI2a-b-c | 18.56 | 7.64 | p=4.55x10^-08^ |
| V. marina BBI3 | -4.98 | -4.04 | p=0.28 |
| V. mungo BBI1 | 15.09 | 3.55 | p=4.43x10^-8^ |
| V. mungo BBI2 | 12.22 | 2.33 | p=5.11x10^-9^ |
| V. mungo BBI3 | 10.09 | -5.51 | p=4.41x10^-9^ |
| V. mungo BBI4 | 3.28 | 9.23 | p=4.81x10^-6^ |
| V. vexillata BBI1 | 8.15 | 5.56 | p=0.0016 |
| V. vexillata BBI2 | 10.48 | 8.08 | p=0.01 |
| V. vexillata BBI3a | 4.04 | 9.78 | p=1.5x10^-8^ |
| V. vexillata BBI3b | 1.78 | 8.4 | p=1.59x10^-6^ |
| V. radiata BBI1 | 8.96 | 3.62 | p=1.49x10^-8^ |
| V. radiata BBI3 | -2.55 | 2.76 | p=2.11x10^-6^ |
| V. unguiculata BBI1 | 12.57 | 3.89 | p=4.13x10^-7^ |
| V. unguiculata BBI2 | 10.05 | 4.45 | p=0.0002 |
| V. unguiculata BBI3a | 1.22 | 5.29 | p=4.09x10^-5^ |
| V. unguiculata BBI3b | 0.15 | 6.87 | p=3.07x10^-11^ |
| V. unguiculata BBI4a-b-c | 2.11 | 8.29 | p=2.57x10^-11^ |

Supplementary Tables 8. Codes of sequences for *BBI1*. ID = sequence name containing taxonomic identification followed by relative accession or genome code and individual number. For heterozygous individuals each sequence was divided in two haplotypes identified as “H1” and “H2”. P=protein identification code, I=Isoform identification code. S=nucleotide identification code. Form (F) = wild (W) or domesticated (D). Origin= species native range (Dachapak et al., 2017; Pootakham et al., 2023; Maxted et al., 2004; Plants of the World Online, Kew Science, ttp://powo.science.kew.org/taxon/urn:lsid:ipni.org:names:325971-2).

| **ID** | **P** | **I** | **S** | **F** | **Origin** |
| --- | --- | --- | --- | --- | --- |
| V.aconitifolia_NI482_1 | 11 | 15 | 16 | D | Asia |
| V.aconitifolia_NI482_2 | 11 | 15 | 16 | D | Asia |
| V.angivensis_NI378_2 | 21 | 14 | 15 | W | Africa |
| V.angivensis_NI378_3 | 21 | 14 | 15 | W | Africa |
| V.angularis_BGE022149_1_H1 | 8 | 1 | 1 | D | Asia |
| V.angularis_BGE022149_1_H2 | 9 | 2 | 2 | D | Asia |
| V.angularis_BGE022149_2_H1 | 8 | 1 | 1 | D | Asia |
| V.angularis_BGE022149_2_H2 | 9 | 2 | 2 | D | Asia |
| V.angularis_GCA_016808095_1 | 9 | 2 | 2 | D | Asia |
| V.angularis_GCA_016808095_1 | 9 | 2 | 2 | D | Asia |
| V.angularis_NI1634_2_H1 | 8 | 1 | 1 | W | Asia |
| V.angularis_NI1634_2_H2 | 9 | 2 | 2 | W | Asia |
| V.angularis_NI1634_3_H1 | 8 | 1 | 1 | W | Asia |
| V.angularis_NI1634_3_H2 | 9 | 2 | 2 | W | Asia |
| V.exilis_NI1926_1 | 18 | 11 | 12 | W | Asia |
| V.exilis_NI1926_2 | 18 | 11 | 12 | W | Asia |
| V.exilis_Vexilis_v1 | 18 | 11 | 12 | W | Asia |
| V.gracilis_TVNU610_1 | 31 | 29 | 32 | W | Africa |
| V.gracilis_TVNU610_2 | 31 | 29 | 32 | W | Africa |
| V.grandiflora_NI1721_1 | 16 | 9 | 10 | W | Asia |
| V.hirtella_GCA_027742795_1 | 9 | 2 | 5 | W | Asia |
| V.hirtella_NI1377_1 | 9 | 2 | 5 | W | Asia |
| V.hirtella_NI1377_2 | 9 | 2 | 5 | W | Asia |
| V.hosei_TVNU1147_1 | 26 | 21 | 22 | W | Africa |
| V.hosei_TVNU1147_2 | 26 | 21 | 22 | W | Africa |
| V.kirkii_TVNU22_2 | 30 | 27 | 29 | W | Africa |
| V.kirkii_TVNU22_3 | 30 | 27 | 29 | W | Africa |
| V.lasiocarpa_TVNU1018_2 | 23 | 17 | 18 | W | America |
| V.lasiocarpa_TVNU1018_3 | 23 | 17 | 18 | W | America |
| V.laurentii_NI1434_1 | 13 | 6 | 7 | W | Africa |
| V.membranacea_ssp._membranacea_NI1448_1 | 21 | 41 | 56 | W | Africa |
| V.membranacea_ssp._membranacea_NI1448_2 | 21 | 41 | 56 | W | Africa |
| V.membranacea_ssp._membranacea_NI1448_3 | 21 | 41 | 56 | W | Africa |
| V.minima_NI1376_1 | 9 | 2 | 5 | W | Asia |
| V.minima_NI1376_2 | 9 | 2 | 5 | W | Asia |
| V.monophylla_NI1564_1 | 15 | 8 | 9 | W | Africa |
| V.monophylla_NI1564_2 | 15 | 8 | 9 | W | Africa |
| V.mungo_BGE040528_1 | 10 | 3 | 3 | D | Asia |
| V.mungo_BGE040528_2 | 10 | 3 | 3 | D | Asia |
| V.mungo_GCA_019096145_1 | 24 | 22 | 23 | D | Asia |
| V.mungo_TVNU1076_2 | 24 | 18 | 19 | W | Asia |
| V.mungo_TVNU1268_2 | 24 | 22 | 23 | W | Asia |
| V.nepalensis_NI1704_1 | 9 | 2 | 5 | W | Asia |
| V.nepalensis_NI1704_2 | 9 | 2 | 5 | W | Asia |
| V.nyangensis_NI1522_1 | 14 | 7 | 8 | W | Africa |
| V.parkeri_NI1969_1 | 19 | 12 | 13 | W | Africa |
| V.parkeri_NI1969_2 | 19 | 12 | 13 | W | Africa |
| V.racemosa_TVNU1521_3 | 26 | 21 | 26 | W | Africa |
| V.racemosa_TVNU973_2 | 26 | 21 | 22 | W | Africa |
| V.racemosa_TVNU973_3 | 26 | 21 | 22 | W | Africa |
| V.radiata_GCA_000741045_2 | 22 | 16 | 17 | D | Asia |
| V.radiata_RC_1 | 22 | 16 | 17 | D | Asia |
| V.radiata_RC_2 | 22 | 16 | 17 | D | Asia |
| V.radiata_TVNU1250_1 | 22 | 16 | 17 | W | Asia |
| V.radiata_TVNU1250_2 | 22 | 16 | 17 | W | Asia |
| V.radicans_NI238_1 | 20 | 13 | 14 | W | Africa |
| V.reflexopilosa_GCA_027742785_1 | 17 | 10 | 11 | D | Asia |
| V.reflexopilosa_GCA_027742785_1 | 32 | 30 | 33 | D | Asia |
| V.reticulata_TVNU1104_2 | 25 | 20 | 21 | W | Africa |
| V.reticulata_TVNU1104_3 | 25 | 20 | 21 | W | Africa |
| V.subramaniana_NI1135_1 | 11 | 4 | 4 | W | Asia |
| V.subramaniana_NI1135_2 | 11 | 4 | 4 | W | Asia |
| V.tenuicaulis_NI1927_1 | 9 | 2 | 2 | W | Asia |
| V.tenuicaulis_NI1927_2 | 9 | 2 | 2 | W | Asia |
| V.trilobata_TVNU1332_1 | 27 | 23 | 24 | W | Asia |
| V.trilobata_TVNU1332_2 | 27 | 23 | 24 | W | Asia |
| V.trinervia_GCA_027743565_1 | 17 | 10 | 11 | W | Asia |
| V.trinervia_NI1737_2 | 17 | 10 | 11 | W | Asia |
| V.trinervia_NI1737_3 | 17 | 10 | 11 | W | Asia |
| V.umbellata_GCA_025174585_1 | 10 | 3 | 3 | D | Asia |
| V.umbellata_var.umbellata_NI21_1 | 10 | 3 | 3 | D | Asia |
| V.umbellata_var.umbellata_NI21_2 | 10 | 3 | 3 | D | Asia |
| V.umbellata_var._gracilis_NI1398_1 | 12 | 5 | 6 | W | Asia |
| V.umbellata_var._gracilis_NI1398_2 | 12 | 5 | 6 | W | Asia |
| V.unguiculata_OR398407_1 | 1 | 19 | 20 | W/D | Africa |
| V.unguiculata_OR398421_1 | 2 | 28 | 47 | D | Africa |
| V.venulosa_TVNU179_1 | 29 | 26 | 28 | W | Africa |
| V.vexillata_TVNU1476_1 | 28 | 24 | 25 | W | Africa |
| V.vexillata_TVNU1476_2 | 28 | 24 | 25 | W | Africa |
| V.vexillata_Vvaxillata_v1 | 28 | 24 | 34 | W | Africa |
| V.wittei_TVNU332_1 | 20 | 13 | 30 | W | Africa |
| V.wittei_TVNU332_2 | 20 | 13 | 30 | W | Africa |

Supplementary Tables 9. Codes of sequences for *BBI2* .ID = sequence name containing taxonomic identification followed by relative accession or genome code and individual number. For heterozygous individuals each sequence was divided in two haplotypes identified as “H1” and “H2”. P=protein identification code, I=Isoform identification code. S=nucleotide identification code. Form (F) = wild (W) or domesticated (D). Origin= species native range (Dachapak et al., 2017; Pootakham et al., 2023; Maxted et al., 2004; Plants of the World Online, Kew Science, http://powo.science.kew.org/taxon/urn:lsid:ipni.org:names:325971-2).

| **ID** | **P** | **I** | **S** | **F** | **Origin** |
| --- | --- | --- | --- | --- | --- |
| V.aconitifolia_NI482_1 | 26 | 18 | 19 | D | Asia |
| V.aconitifolia_NI482_2 | 26 | 18 | 19 | D | Asia |
| V.ambacensis_TVNU1150_1 | 32 | 26 | 27 | W | Africa |
| V.ambacensis_TVNU1150_2 | 32 | 26 | 27 | W | Africa |
| V.angivensis_NI378_2 | 24 | 16 | 17 | W | Africa |
| V.angivensis_NI378_3 | 24 | 16 | 17 | W | Africa |
| V.angularis_GCA_016808095_1 | 14 | 7 | 7 | D | Asia |
| V.angularis_GCA_016808095_1 | 14 | 7 | 7 | D | Asia |
| V.angularis_NI1634_2 | 14 | 7 | 7 | W | Asia |
| V.angularis_NI1634_3 | 14 | 7 | 7 | W | Asia |
| V.dolomitica_NI2203_1 | 22 | 14 | 15 | W | Africa |
| V.exilis_NI1926_1 | 11 | 10 | 10 | W | Asia |
| V.exilis_NI1926_2 | 11 | 10 | 10 | W | Asia |
| V.exilis_Vexilis_v1 | 11 | 10 | 10 | W | Asia |
| V.gracilis_TVNU610_1 | 38 | 34 | 38 | W | Africa |
| V.gracilis_TVNU610_2 | 38 | 34 | 38 | W | Asia |
| V.heterophylla_NI1485_1 | 16 | 6 | 6 | W | Africa |
| V.heterophylla_NI1485_2 | 16 | 6 | 6 | W | Africa |
| V.hirtella_GCA_027742795_1 | 14 | 4 | 4 | W | Asia |
| V.hirtella_NI1377_1 | 14 | 4 | 4 | W | Asia |
| V.hirtella_NI1377_2 | 14 | 4 | 4 | W | Asia |
| V.kirkii_TVNU22_2_H1 | 35 | 31 | 33 | W | Africa |
| V.kirkii_TVNU22_2_H2 | 35 | 31 | 34 | W | Africa |
| V.kirkii_TVNU22_3 | 35 | 31 | 34 | W | Africa |
| V.kirkii_TVNU364_2 | 35 | 31 | 75 | W | Africa |
| V.kirkii_TVNU364_3 | 35 | 31 | 75 | W | Africa |
| V.lanceolata_NI1860_2 | 18 | 9 | 9 | W | Oceania |
| V.lasiocarpa_TVNU1018_1 | 28 | 21 | 22 | W | America |
| V.lasiocarpa_TVNU1018_2 | 28 | 21 | 22 | W | America |
| V.lasiocarpa_TVNU1018_3 | 28 | 21 | 22 | W | America |
| V.laurentii_NI1434_1 | 15 | 5 | 5 | W | Africa |
| V.laurentii_NI1434_2 | 15 | 5 | 5 | W | Africa |
| V.luteola_TVNU487_2 | 36 | 32 | 35 | W | Africa |
| V.marina_TVNU1441_1 | 33 | 28 | 30 | W | Africa |
| V.marina_Vmarina_v11 | 36 | 35 | 39 | W | Africa |
| V.marina_Vmarina_v12 | 33 | 36 | 40 | W | Africa |
| V.marina_Vmarina_v13 | 33 | 36 | 41 | W | Africa |
| V.membranacea_ssp._caesia_TVNU890_2 | 21 | 13 | 14 | W | Africa |
| V.membranacea_ssp._caesia_TVNU897_3 | 21 | 13 | 14 | W | Africa |
| V.membranacea_ssp._hapalantha_TVNU146_1 | 21 | 13 | 14 | W | Africa |
| V.membranacea_ssp._hapalantha_TVNU146_2 | 21 | 13 | 14 | W | Africa |
| V.minima_NI1376_1 | 14 | 4 | 4 | W | Asia |
| V.minima_NI1376_2 | 14 | 4 | 4 | W | Asia |
| V.mudenia_NI2152_1 | 21 | 13 | 13 | W | Africa |
| V.mudenia_NI2152_2 | 21 | 13 | 13 | W | Africa |
| V.mungo_BGE040528_1 | 11 | 1 | 1 | D | Asia |
| V.mungo_BGE040528_2 | 11 | 1 | 1 | D | Asia |
| V.mungo_GCA_019096145_1 | 29 | 22 | 28 | D | Asia |
| V.mungo_TVNU1076_1 | 29 | 22 | 23 | W | Asia |
| V.mungo_TVNU1076_2 | 29 | 22 | 23 | W | Asia |
| V.mungo_TVNU1268_2 | 29 | 22 | 28 | W | Asia |
| V.nepalensis_NI1704_1 | 17 | 8 | 8 | W | Asia |
| V.nepalensis_NI1704_2 | 17 | 8 | 8 | W | Asia |
| V.oblongifolia_var.oblongifolia_NI461_1 | 25 | 17 | 18 | W | Africa |
| V.oblongifolia_var.oblongifolia_NI461_2 | 25 | 17 | 18 | W | Africa |
| V.parkeri_NI1969_1 | 19 | 11 | 11 | W | Africa |
| V.parkeri_NI1969_2 | 19 | 11 | 11 | W | Africa |
| V.pseudovenulosa_NI410_2 | 43 | 60 | 76 | W | Africa |
| V.pubigera_var.pubigera_NI1147_2 | 31 | 25 | 26 | W | Africa |
| V.racemosa_TVNU973_2 | 13 | 3 | 36 | W | Africa |
| V.racemosa_TVNU973_3 | 13 | 3 | 36 | W | Africa |
| V.radicans_NI238_1 | 23 | 15 | 16 | W | Africa |
| V.reflexopilosa_GCA_027742785_1 | 39 | 37 | 42 | W | Asia |
| V.reflexopilosa_GCA_027742785_1 | 40 | 38 | 43 | D | Asia |
| V.reticulata_TVNU1104_1 | 37 | 33 | 37 | W | Africa |
| V.reticulata_TVNU1104_2 | 37 | 33 | 37 | W | Africa |
| V.stipulacea_NI1030_2 | 12 | 2 | 2 | W | Asia |
| V.subterranea_TVSU1295_1 | 20 | 12 | 12 | D | Africa |
| V.subterranea_TVSU305_1 | 20 | 12 | 12 | D | Africa |
| V.subterranea_var._spontanea_NI2008_1 | 20 | 12 | 12 | W | Africa |
| V.subterranea_var._spontanea_NI2008_2 | 20 | 12 | 12 | W | Africa |
| V.subterranea_Vigsu_genome | 20 | 39 | 44 | D | Africa |
| V.tenuicaulis_NI1927_1 | 17 | 8 | 8 | W | Asia |
| V.trilobata_TVNU1332_1 | 29 | 27 | 29 | W | Asia |
| V.trilobata_TVNU1332_2 | 29 | 27 | 29 | W | Asia |
| V.trinervia_GCA_027743565_1 | 39 | 37 | 42 | W | Asia |
| V.umbellata_GCA_025174585_1 | 11 | 1 | 1 | D | Asia |
| V.umbellata_var.umbellata_NI21_1 | 11 | 1 | 1 | D | Asia |
| V.umbellata_var.umbellata_NI21_2 | 11 | 1 | 1 | D | Asia |
| V.umbellata_var._gracilis_NI1398_1 | 11 | 20 | 21 | W | Asia |
| V.umbellata_var._gracilis_NI1398_2 | 11 | 20 | 21 | W | Asia |
| V.umbellata_var._gracilis_NI1398_3 | 11 | 20 | 21 | W | Asia |
| V.unguiculata_OR398431_1 | 1 | 41 | 46 | W/D | Africa |
| V.unguiculata_OR398434_1 | 3 | 43 | 49 | W | Africa |
| V.vexillata_TVNU1476_1 | 34 | 29 | 31 | W | Africa |
| V.vexillata_TVNU1476_2 | 34 | 29 | 31 | W | Africa |
| V.vexillata_Vvaxillata_v1 | 42 | 42 | 47 | W | Africa |

Supplementary Tables 10. Ancestral Isoform of BBI1 and their mature protein.

| **Ancestral isoform** | **mature protein (modern isoform)** |
| --- | --- |
| AncI0 | AncP1 |
| AncI1 | AncP2 |
| AncI2 | P9 (I02) |
| AncI3 | AncP3 |
| AncI4 | P24 (I18, I22) |
| AncI5 | P1 (I19) |
| AncI6 | P28 (I24) |
| AncI7 | AncP4 |

Supplementary Tables 11. Ancestral Isoform of BBI2 and their mature protein.

| **Ancestral isoform** | **mature protein (modern isoform)** |
| --- | --- |
| AncI0 | AncP1 |
| AncI1 | AncP2 |
| AncI2 | AncP3 |
| AncI3 | P39 (I37) |
| AncI4 | P24 (I16) |
| AncI5 | AncP4 |
| AncI6 | P3 (I43) |
| AncI7 | AncP5 |
| AncI8 | P20 (I12, I39) |

Supplementary Tables 12. Summary of results of the positive selection tests. Both genes show positive selection according to the results of the Site Model test (M8a vs. M8). FUBAR, MEME, NEB and BEB analysis were applied to identify the codons targeted by positive selection. Position of residue for each amino acid alignment is reported if *P* ≥0.95 for FUBAR, NEB and BEB and if p≤0.05 for MEME analysis. The residues that determines the interaction with the target (P_1_) for both domains are in bold and the letters indicate the amino acids involved.

|  | **FUBAR** | **MEME** | **NEB** | **BEB** |
| --- | --- | --- | --- | --- |
| *BBI1* | 32, P=1.00; 54, P=0.98; **59, K/R/A, P=0.99**; 98, P=0.96; 108, P=0.98; | 14, p=0.02; 39, p=0.02; **59, K/R/A, p<0.001**; **86, A/R p<0.001** | 32, P=1.00, ω= 2.76; **59, K/R/A, P=0.99,** ω**=2.74**; | 32, H P=1.00, ω=2.39; **59, K/R/A, P=0.99,** ω**=2.38**; |
| *BBI2* | 55, P=0.98; 57, P=0.97; **60, R/K/H/E, P=0.99**; 84, 0.96; **87, Y/F/L, P=0.97;** | 57, p=0.02; **60, R/K/H/E, p=0.01** | 28, Q P=0.96, ω=3.41; 55, E, P=0.96, ω=3.41; **60, R/K/H/E, P=1.00,** ω**=3.52**; 84, A, P=1.00, ω=3.52; 113 D, P=1.00, ω=3.52 | 28 Q P=0.99, ω=2.82; 55, E, P=0.99, ω=2.82; **60, R/K/H/E, P=1.00,** ω**=2.84**; 84, A, P=1.00, ω=2.84; 113 D, P=1.00, ω=2.84 |

Supplementary Tables 13. Phylogenetic trees in newick format produced by IQtree. Include length of branches and support values on the nodes (SH-aLRT/Approximate Bayes Test/Ultrafast bootstrap).

| **Alignments** | **Phylogenetic trees** |
| --- | --- |
| amino acid sequences (BBI1) | (I01:0.0097265016,(I02:0.0000020302,((((I04:0.0094545977,I16:0.0095871700)88.1/0.996/95:0.0189057596,(I09:0.0404786408,I15:0.0096005788)75.7/0.747/58:0.0089915525)85.9/0.999/70:0.0193399268,(((((((((((I06:0.0170047694,I12:0.0000020302)85.6/0.999/92:0.0275243304,I29:0.0769388641)85/0.946/95:0.0249434866,I21:0.0312460999)90.9/1/95:0.0376453779,I17:0.0624536838)69.9/0.429/85:0.0087354297,I27:0.0682738850)85.1/0.948/90:0.0219551496,(I07:0.0096882806,I08:0.0000020302)77.7/0.896/93:0.0096823308)87.9/1/87:0.0215362186,(((I13:0.0630744258,I20:0.0417447003)92.6/1/92:0.0416809479,(I19:0.0093347718,I28:0.0093663856)0/0.333/51:0.0000020302)80.1/0.847/82:0.0112857274,((I14:0.0000020302,I41:0.0184845175)74.8/0.702/92:0.0089279808,I24:0.0191502274)95.2/1/98:0.0438654374)76/0.563/70:0.0095818747)86.5/0.997/93:0.0242257155,I26:0.0620109629)92.9/1/95:0.0409964692,I23:0.0187510933)0/0.333/44:0.0000020302,(I18:0.0000020302,I22:0.0091640085)98.2/1/99:0.0522033479)77/0.979/61:0.0092703556,I10:0.0094063620)0/0.333/42:0.0000020302)99.9/1/100:0.1128327503,I05:0.0515784826)0/0.333/44:0.0000020302)0/0.333/39:0.0000020302,((I03:0.0099806982,I11:0.0192920231)94.6/1/96:0.0405144452,I30:0.0197578718)0/0.333/62:0.0000020302); |
| amino acid sequences (BBI2) | (I01:0.0000020302,((((((I02:0.0316158103,I27:0.0103032552)0/0.333/45:0.0000020302,I22:0.0000020302)0/0.333/59:0.0000020302,I37:0.0101682266)0/0.333/46:0.0000020302,I18:0.0426672877)96.3/1/100:0.0691257929,((((I03:0.0336480385,((I05:0.0172312432,I11:0.0207583183)84/0.955/98:0.0271626238,I34:0.0412529788)79.7/0.997/86:0.0515310936)65.9/0.977/81:0.0508485855,(((((I06:0.0108000737,(I25:0.0000020302,I26:0.0102215772)76.9/0.7/99:0.0090174709)92.9/1/89:0.0299150766,((((I17:0.0495961558,I28:0.0095287492)0/0.333/36:0.0000021522,I35:0.0000020302)91.5/0.999/67:0.0095619980,I32:0.0000020302)0/0.333/33:0.0000020302,I36:0.0095334005)75.7/0.707/75:0.0093242482)64/0.975/71:0.0194727733,(I12:0.0000020302,I39:0.0095337494)92.9/1/97:0.0293679668)0/0.333/65:0.0000020302,I60:0.0197145476)74.8/0.885/83:0.0106066266,I09:0.0482501945)83.6/0.99/87:0.0379168951)89.4/0.999/82:0.0416224134,((I13:0.0261284850,(I14:0.1452170908,(I15:0.0129264575,I33:0.0440887004)75.1/0.941/95:0.0382431204)93.8/1/99:0.0617934479)79.4/0.997/86:0.0431915285,(I21:0.1087362989,I31:0.0105175661)85.1/0.984/76:0.0227090766)77.2/0.644/39:0.0234149812)71.5/0.477/56:0.0137904627,((I16:0.0100963143,(I29:0.0000020302,I42:0.0101903684)78.1/0.96/98:0.0103345102)95.9/1/100:0.0669199550,(I41:0.0194111290,I43:0.0000020788)98.1/1/100:0.0877546885)93.4/1/97:0.0680827345)90.1/0.999/98:0.0445479509)98.6/1/100:0.1024916878,((I04:0.0000020302,I08:0.0100447103)0/0.333/56:0.0000020070,I07:0.0101629530)76.4/0.913/96:0.0113692526)74.5/0.544/88:0.0089535012,((I10:0.0102050987,I38:0.0102393234)73.7/0.372/86:0.0062763734,I20:0.0086084381)73.6/0.359/85:0.0053148987); |

# References

Dachapak, S., Somta, P., Poonchaivilaisak, S., Yimram, T., and Srinives, P. (2017). Genetic diversity and structure of the zombi pea (*Vigna vexillata* (L.) A. Rich) gene pool based on SSR marker analysis. *Genetica* 145, 189–200. doi: 10.1007/s10709-017-9957-y

Emms, D. M., and Kelly, S. (2019). OrthoFinder: phylogenetic orthology inference for comparative genomics. *Genome Biol* 20, 238. doi: 10.1186/s13059-019-1832-y

Hall T. A. (1999). BioEdit: a user-friendly biological sequence alig nment editor and analysis program for Windows 95/98/NT. *Nucleic Acids Symp Ser*. (London: Retrieval Ltd), 95–98.

Maxted, N., Mabuza-Diamini, P., Moss, H., Padulosi, S., Jarvis, A., and Guarino, L. (2004). *An ecogeographic study: African Vigna, systematic and ecogeographic studies of crop genepool 10*. Rome, Italy: International Plant Genetic Resources Institutes.

Pootakham, W., Sonthirod, C., Naktang, C., Yundaeng, C., Yoocha, T., Kongkachana, W., et al. (2023). Genome assemblies of *Vigna reflexo-pilosa* (créole bean) and its progenitors, *Vigna hirtella* and *Vigna trinervia*, revealed homoeolog expression bias and expression-level dominance in the allotetraploid. *GigaScience* 12, giad050. doi: 10.1093/gigascience/giad050

Untergasser, A., Cutcutache, I., Koressaar, T., Ye, J., Faircloth, B. C., Remm, M., et al. (2012). Primer3—new capabilities and interfaces. *Nucleic Acids Res* 40, e115. doi: 10.1093/nar/gks596

*Vigna* Savi | Plants of the World Online | Kew Science (n.d.). Plants of the World Online. Available at: http://powo.science.kew.org/taxon/urn:lsid:ipni.org:names:325971-2 (Accessed on 2024).
